# Dissecting Genetic Networks Underlying Complex Phenotypes: The Theoretical Framework

**DOI:** 10.1371/journal.pone.0014541

**Published:** 2011-01-20

**Authors:** Fan Zhang, Hu-Qu Zhai, Andrew H. Paterson, Jian-Long Xu, Yong-Ming Gao, Tian-Qing Zheng, Rong-Ling Wu, Bin-Ying Fu, Jauhar Ali, Zhi-Kang Li

**Affiliations:** 1 Institute of Crop Sciences/National Key Facility for Crop Gene Resources and Genetic Improvement, Chinese Academy of Agricultural Sciences, Beijing, China; 2 Plant Genome Mapping Laboratory, University of Georgia, Athens, Georgia, United States of America; 3 Center for Statistical Genetics, Pennsylvania State University, Hershey, Pennsylvania, United States of America; 4 Plant Breeding, Genetics, and Biotechnology Division, International Rice Research Institute, Manila, Philippines; University of Umeå, Sweden

## Abstract

Great progress has been made in genetic dissection of quantitative trait variation during the past two decades, but many studies still reveal only a small fraction of quantitative trait loci (QTLs), and epistasis remains elusive. We integrate contemporary knowledge of signal transduction pathways with principles of quantitative and population genetics to characterize genetic networks underlying complex traits, using a model founded upon one-way functional dependency of downstream genes on upstream regulators (the principle of hierarchy) and mutual functional dependency among related genes (functional genetic units, FGU). Both simulated and real data suggest that complementary epistasis contributes greatly to quantitative trait variation, and obscures the phenotypic effects of many ‘downstream’ loci in pathways. The mathematical relationships between the main effects and epistatic effects of genes acting at different levels of signaling pathways were established using the quantitative and population genetic parameters. Both loss of function and “co-adapted” gene complexes formed by multiple alleles with differentiated functions (effects) are predicted to be frequent types of allelic diversity at loci that contribute to the genetic variation of complex traits in populations. Downstream FGUs appear to be more vulnerable to loss of function than their upstream regulators, but this vulnerability is apparently compensated by different FGUs of similar functions. Other predictions from the model may account for puzzling results regarding responses to selection, genotype by environment interaction, and the genetic basis of heterosis.

## Introduction

Great progress has been made in genetic dissection of quantitative trait variation during the past two decades, but a few puzzling results have recurred in many QTL mapping studies. First, only a small fraction of QTLs are detectable in any one study, regardless of the complexity of traits and test environments [1]–[3]. Second, few QTLs are detected with large and consistent effects. Third, epistasis has been elusive, although increased power and accuracy in QTL detection show it to contribute substantially to complex inheritance in plants [4]–[19], usually occurring between complementary loci [4]–[8]. Fourth, QTLs having large additive effects and those having non-additive effects appear to behave differently in both dominance and epistasis [5]–[11]. Fifth, an increasing number of large-effect QTLs have been cloned in plants and animals and in most these cases, the large phenotypic effects were attributed to the differences between a functional (expressed) allele and a loss of function mutant (Table S1). Finally, recent mapping studies were able to detect more QTLs of small effect using large populations [20], [21], but epistasis between or among QTLs were not adequately addressed in these studies and phenotyping large populations poses a tremendous challenge, particularly across multiple environments.

In parallel with progress in genetic dissection of quantitative traits, molecular studies have shown that biological processes of multicellular organisms and their responses to external cues are controlled by complex gene networks consisting of multiple hierarchical signaling pathways [22], [23]. For example, small groups of signal transduction (**S**) genes functioning in perception and response to specific internal or external cues may initiate expression of larger groups of genes acting in transcriptional and post-transcription regulation (**T**) of still larger numbers of downstream genes in various biochemical pathways (**B**) that ultimately affect phenotype. Thus, there are clear functional relationships between and among genes acting within a signaling pathway by molecular mechanisms such as protein-protein, protein-DNA and protein-RNA interactions, etc (Fig. S1).

While gene networks controlling biological processes presumably include the genetic determinants of complex trait variation, these two important areas of study have remained largely independent. For example, gene networks consisting of multiple hierarchical signaling pathways might explain high-order epistasis, but only digenic epistasis affecting complex traits has been possible to map [24], [25]. Recent modeling efforts have suggested that epistasis might be better explained by functional relationships in regulatory networks [26] than the classical genetics models for qualitative traits [27] and inclusion of the non-linear epistatic interactions and environmental factors in the genetics model can significantly improve the accuracy in predicting the genotype-phenotype relationships of complex traits [28]–[30]. However, it remains challenging to link the functional dependency among genes in signaling pathways with statistical epistasis detected at the phenotypic level. For example, how can hierarchical and non-hierarchical relationships among members of gene networks underlying complex traits be distinguished from one another based on estimated QTL parameters? Moreover, what function(s) and level(s) in the hierarchy most frequently account for allelic variation giving rise to QTLs?

We describe a model that integrates contemporary knowledge of signal transduction pathways with principles of quantitative and population genetics to form a theoretical framework for improved understanding of the genetic control of complex traits. As examples, the model is applied to study putative genetic networks affecting two complex traits of rice, plant height (PH) and submergence tolerance (ST), suggesting strong links between complex phenotypes and their underlying genes.

### The molecular and quantitative genetics framework

**Figure 1:**
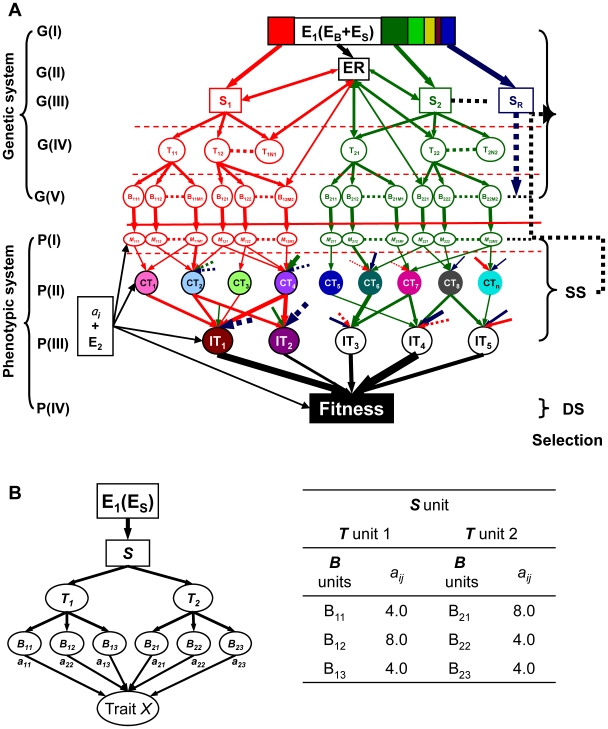
Molecular quantitative genetics models underlying expression of complex traits. (**A**) A generalized molecular quantitative genetics **model (1)** underlying expression of complex traits. **E_1_**, ***S***, ***ER***, ***T*** and ***B*** represent five major levels {G(I) - G(V)} of the genetic system at the **s**ignal transduction regulatory level, **e**pigenetic **r**egulatory level, **t**ranscriptional-posttranscriptional regulatory level, and **b**iosynthesis-transportation level. P(I), P(II), P(III) and P(IV) represent the four levels of the phenotypic system with P(I)  =  metabolites at the biochemical level (M_ijk_), P(II)  =  component traits (CTs), P(III)  =  integrated traits (ITs), and P(IV)  =  fitness. SS and DS represent the two major types of selection - the stabilizing selection and directional selection defined in the population genetics theory. **E_1_** and **E_2_** represent two types of environmental components. **E_1_** represents the physical environment of a multicellular organism encounters, which contains two parts, **E_B_** (the basic or average elements in an environment required for the organism to survive) and **E_S_** (the unique physical features of the environment that deviate from **E_B_** and require expression of specific signaling pathways for survival). Thus, **E_1_** is part of the genetic system. **E_2_** is the random and non-heritable part of any phenotypes measured in the environment [31]–[33]; (**B**) A simplified molecular quantitative genetics **model (2)** of a single signaling pathway consisting of a single ***S*** unit, 2 ***T*** units, and 6 downstream ***B*** units underlying expression of trait ***X***.

Figure 1A shows a generalized **model (1)** of the relationship between underlying genes and complex phenotypes of a multicellular organism in a specific environment. This model has two major parts, the genetic system (or gene networks) and phenotypic system.

The genetic system of **model (1)** can be divided into five major components:

- G(I): **E_1_**, the physical environment, subdivided into **E_B_** (the basic requirements for an organism to live) and **E_S_** (specific and unique physical/biological elements of the environment that deviate from **E_B_**), that requires expression of specific signaling pathways for the organism to acclimate or adapt;
- G(II): the epigenetic regulatory element (***ER***);
- G(III): the signal transduction regulatory element (***S***);
- G(IV): the transcriptional and post-transcriptional regulatory element (***T***); and
- G(V): the biosynthesis and transport element (***B***).

In **model (1)**, the genetic system can be conceptualized as a complex network consisting of multiple signaling pathways, each with a single ***S*** unit regulating multiple ***T*** and downstream ***B*** units. Genes involved in levels ***S***, ***T*** and ***B*** of the genetic network underlying a single signaling pathway have different but well defined functions, as briefly described above.

**The phenotypic system of model (1)** can also be conceptualized as 4 related layers:

- P(I): metabolites (**M**) or biochemical traits;
- P(II): component traits (**CT**);
- P(III): integrated traits (**IT**); and
- P(IV): fitness, defined here as differential survival and reproduction.

Any measurable phenotype may be affected by genes at any level of a network. To better illustrate the relationships between the genetic system of **model (1)** and their resulting phenotypes, we tentatively define a group of functionally dependent genes acting at each level of a signaling pathway as a functional genetic unit (**FGU**) with functional alleles of all constituent loci required for the FGU to function normally and have an effect (*a_ij_*) on phenotype (Table S2). When one or more of its member genes or regulators are nonfunctional, an FGU may have no phenotypic effect.

**Model (1)** has two different environmental elements, **E_1_** and **E_2_**. **E_1_** corresponds to the conventional ‘macro-environment’, which contains two components, **E_B_** and **E_S_**. **E_S_** accounts for the genotype x environment (GE) interaction observed in almost all complex phenotypes of multicellular organisms. **E_2_** is the random and non-heritable part of a phenotype measured in an environment that is a major component of trait heritability defined in classical quantitative genetics theory [31]–[33]. Thus, a phenotype (CT_i_, IT_j_ or fitness) measured in a specific environment is the consequence of the interaction between the genetic system and **E_2_**.

### The principle of hierarchy

Molecular genetic studies indicate a general one-way functional dependency of downstream loci on their upstream regulators. Thus, each signaling pathway in the genetic network of **model (1)** can be envisioned as having a single ***S*** unit at the top, multiple ***T*** units in the middle, and their regulated ***B*** units such that downstream FGUs are always dependent on functionality of an upstream FGU. Upstream FGUs are generally few in number and relatively conserved evolutionarily, with larger phenotypic effects than downstream units. This **principle of hierarchy** in gene number, diversity, and phenotypic effect is the foundation upon which the theory and methodology for detecting genetic networks underlying complex phenotypes can be developed.

### Computer simulation

#### Simulation (1)

The genetic network of **model (1)** consists of multiple signaling pathways, each of which contains FGUs in three major layers specified as **model (2)** (Fig. 1B, Table 1). In **model (2)**, a quantitative trait, ***X***, is affected by a single signaling pathway consisting of 3 FGU levels - a single ***S*** element that regulates 2 ***T*** (***T_1_*** and ***T_2_***) elements, each of which controls 3 downstream ***B*** elements which have phenotypic effects of 4 or 8 units on trait ***X***. As described above, all genes within an FGU at any level of **model** **(2)** are functionally dependent on one another, and there is one-way functional dependency (FD) of any gene in a downstream unit on genes in their upstream unit.

**Table 1:** Seven different scenarios under which QTL parameters (main and epistatic effects), in addition to 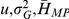, to be simulated in ideal F_2_ and RI (DH) populations regarding the number of segregating loci in different layers of a hypothetical signaling pathway defined in model (2) containing 9 FGUs, 1 ***S*** unit, 2 ***T*** (***T_1_*** and ***T_2_***) units and 6 downstream ***B*** (***B_11_***, ***B_12_***, ***B_13_***, ***B_21_***, ***B_22_***, and ***B_23_***) units that affect a complex trait, ***X*** (Fig. 1B).

Scenario	Number (*r*) of segregating loci at different layers of model (2)^1^									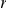	Number of FD^3^		N_1_	Population size (N_2_)^4^		Simulated QTL parameters
	*S*	*T_1_*	*B_11_*	*B_12_*	*B_13_*	*T_2_*	*B_21_*	*B_22_*	*B_23_*		I	II		RI	F_2_	
**1**	0	1	0	0	0	1	0	0	x	2	0	0	2	4	9	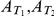
**2**	0	0	1	0	1	0	1	0	1	4	0	0	4	16	81	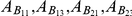
**3**	0	1	1	0	1	1	1	0	1	6	4	0	10	64	729	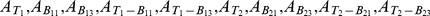
**4**	1	1	1	0	1	1	1	0	1	7	14	0	21	128	2187	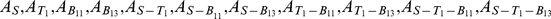 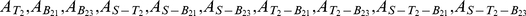
**5**	1	2	0	0	0	3	0	0	0	6	10	5	21	64	729	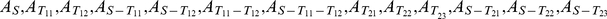 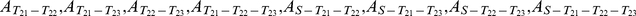
**6**	0	1	2	0	0	1	3	0	0	7	10	5	22	128	2187	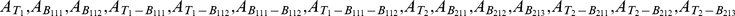 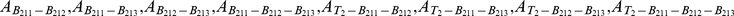
**7**	0	0	0	1	1	1	3	1	1	8	9	4	21	256	6561	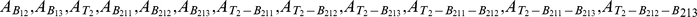 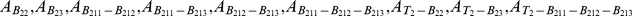
**Effect** 2	32	16	4	8	4	16	8	4	4							

1 ‘0’ in the cells indicates the situations where parents are not segregating and have the same functional alleles at all loci within the unit (the unit is functional), whereas ‘x’ indicates the situation where both parents share the same mutant allele(s) at one or more loci within the unit such that the unit is non-functional (the segregating locus or loci in the parents are non-complementary). 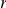 is the total number of the segregating loci in the populations.

2 “Effect” is the maximum pathway phenotypic value on trait ***X*** of the corresponding ***S***, ***T*** and ***B*** loci (or units) specified in **model (2)** (Fig. 1B).

3 FD is functional dependency. There are two types of FD: (I) functional dependency of the downstream FGU loci on loci of their upstream FGUs, and (II) mutual functional dependency of loci from one another in the same FGUs.

4 N_1_ is the total number of genetic parameters simulated in the populations; N_2_ is the population size of the simulated populations and the expected number of possible multilocus genotypes in each simulated population under Hardy-Weinberg equilibrium.

Three important questions arise: (1) how does segregation at different loci in the genetic system of **model (2)** affect trait mean, variation and heterosis in biparental populations; (2) at which levels in **model (2)** can allelic differences be detected as QTLs by conventional quantitative genetics, and (3) if they can all be detected as QTLs, in what ways do loci at different levels of a genetic system differ from one another?

To answer these questions, we simulated 7 scenarios under **model (2)** with positive regulation (i.e. activation by regulatory genes at levels ***S*** and ***T***) in which different numbers of loci in the signaling pathway are segregating in bi-parental populations with 2 alleles of one functional and one non-functional mutant at each segregating locus (Table 1). Two types of functional relationships exist between or among loci within **model (2)**, each of which mimic well known molecular mechanism(s). Type **I** is the one-way FD between downstream loci on their upstream regulatory ones mostly through protein-DNA interactions, while type **II** is the mutual FD mostly through protein-protein interactions and enzyme cooperativity (Fig. S1, [34]). Scenarios 1 and 2 represent the typical assumption of classical quantitative genetics theory that segregating upstream or downstream loci (respectively) are functionally and genetically independent from one another. In scenarios 3 and 4, a single locus at each of 2 (***T*** and ***B***) and 3 levels (***S***, ***T*** and ***B***) of the signaling pathway is segregating such that only type **I** FD exists between a downstream locus and its upstream one. In scenarios 5–7 represent more complicated situations in which one or more loci at 2 (***S*** and ***T***) and 3 levels (***S***, ***T*** and ***B***) of the signaling pathway are segregating such that both types I and II FD exist between downstream and upstream loci, and between different segregating loci within FGUs. Together, these 7 scenarios cover most cases in which epistasis may arise from different FD relationships between loci in a signaling pathway. The specified segregating loci are complementary to one another in the sense that functional genotypes can be generated from recombination of the parental alleles, except in scenario 1 in which the parents had the same mutant allele in unit ***B_23_***. To simulate expected mid-parent heterosis (*H*_MP_), all possible parental genetic compositions at segregating loci were considered equally for each specified scenario, and the expected values of F_1_ and *H*_MP_ were calculated under 3 classical modes of gene action [31] – complete dominance (D) at all segregating loci, additivity (A) at all loci, and a mixed mode of gene action (DA) where regulatory loci at levels ***S*** and ***T*** are assumed to be completely dominant while those at level ***B*** are additive. The simulated populations are ideal in that segregating loci are unlinked, alternative alleles at each locus have frequencies of 0.5, all possible multilocus genotypes occur at the expected Hardy-Weinberg frequencies and linkage equilibrium, phenotypic values of trait ***X*** defined in **model (2)** have 100% penetrance and expressivity, and effects of different ***B*** units are additive. The number of the expected genetic parameters of the segregating loci in each scenario (main and epistatic effects) ranges from 2 in scenario 1 to 22 in scenario 6, which were estimated using conventional QTL methodology [24], [25]. The mathematical relationships between the estimated QTL main and epistatic effects of loci at ***S***, ***T*** and ***B*** levels of **model (2)** and their corresponding pathway effects were derived based on their expectations from the simulated results.

#### Simulation (2)

Further, we estimated frequency shifts (FSs) of individual segregating loci and gametic linkage disequilibria (LDs) between segregating loci resulting from step-wise directional selection towards either increased or decreased trait values under each scenario using the Bennett's method [35]. The FSs at each locus, pairwise LDs between segregating loci, selection intensity, population mean, and genotypic variance at each step of selection were calculated.

## Results

### Theoretical expectations of heterosis and population parameters

Figure S2 shows the phenotypic distributions of multilocus genotypes under the 7 simulated scenarios together with their population mean and variances. Segregation at levels ***S*** and/or ***T*** (scenarios 1, 4 and 5) of **model (2)** results in a typical bimodal phenotypic distribution in the progeny, a greatly reduced trait mean (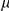) and increased trait variation regardless of the type of gene action. In contrast, largely continuous phenotypic distributions are observed for scenarios 2, 3, 6 and 7 where many ***B*** loci are segregating. Fig. S3 and Table S3 show the expected levels and variation of trait heterosis for each of the 7 scenarios under 3 types of gene action. The direction of trait heterosis is determined by dominance at the regulatory FGUs (***S*** and ***T***). Contributions of segregating loci to the level of heterosis follow the principle of hierarchy that ***S*** loci > ***T*** loci > ***B*** loci.

Epistasis contributes greatly to the level of trait heterosis, independent of gene action, and varies with the degree of functional complementarity, number, and allelic distribution of segregating loci in the parents. Across scenarios 3–7, epistasis and dominance of both regulatory (***S***, ***T***) and downstream (***B***) loci contribute roughly equally to trait heterosis. The correlation between mean heterosis and inbreeding depression is −0.974 under complete dominance, −0.540 under additivity and 0.856 under the mixed mode of gene action. Zero trait heterosis occurs only in the 10 cases of scenarios 1, 2 (no epistasis), even though mean heterosis is virtually zero in all scenarios under additivity. In other words, within a single signaling pathway, negative heterosis results only from additive epistasis (Fig. S3).

### Genetic expectations of QTL parameters

Table S4 and Figure S4 show the expected QTL effects of different segregating loci estimated in the simulated populations using classical QTL mapping methodology [24], [25] and their expected frequency shifts from directional selection in the 7 scenarios, which led us to three important theorems.

#### Theorem 1

In the genetic system of a signaling pathway of **model (2)** with a single upstream ***S*** unit, 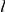 ***T*** units, each of which regulates 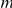 genetically independent downstream ***B*** units with equivalent effects (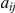) on a complex trait, ***X***, when only a single locus within one or more of the ***B*** units, or ***T*** units, or their upstream ***S*** unit, is segregating in a biparental population (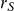, or 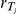, or 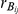 = 1), then the segregating ***B***, ***T*** or ***S*** locus will be detected as a main-effect QTL with an effect equal to its expectation, i.e. one half of its unit effect defined in **model (2)** (scenarios 1 and 2). Here, the functional genotypes are defined as individuals that are either homozygous for the functional allele or heterozygous at the corresponding locus in each of the ***S***, ***T*** and ***B*** units of **model (2)**.

#### Theorem 2

In a signaling pathway of **model (2)**, when 2 or more loci within each level or at different levels are segregating in a biparental population, segregating loci in different ***B*** units within the same or different ***T*** units are genetically independent from one another (no epistasis). Only 2 types of epistasis exist based on functional relationships between or among loci (Table S4): epistasis between alleles at upstream loci and their regulated downstream loci (type **I** in scenarios 3, 4), and epistasis between alleles at different segregating loci within the same unit in each level of the system (type **II** in scenarios 5–7). In these cases, the number and type of digenic and high-order epistasis can be predicted by the number of loci segregating at different levels of the system, 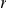, and FD between or among the segregating loci (Tables 1, S4). The relationships between the QTL main additive effects (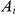) of segregating loci at any level of the signaling pathway and the expected pathway effects (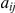) of the FGUs defined in **model (2)** can be described by the general formula in Table 2:
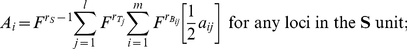
(1)

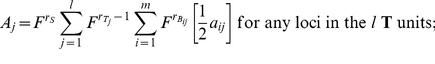
(2)

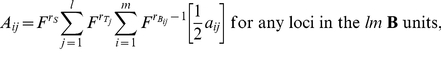
(3)where 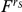, 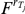 and 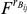 are the frequencies of the functional genotypes at the ***S***, ***T*** and ***B*** units respectively, which is 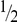 for a RI (or DH) population and 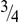 for an F_2_ population (assuming complete dominance at all loci involved), 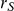, 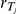 and 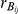
are the numbers of segregating loci at the corresponding ***S***, ***T*** and ***B*** units of the network. In cases of mixed gene action (additivity for all ***B*** loci and complete dominance for the ***S*** and ***T*** loci), the above formulae remain valid except that 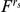 and 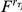 are 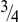, and 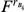 is 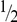.

**Table 2:** Formula for estimating pathway effects (*a_ij_*) based on QTL additive and epistatic effects (*A*) and their corresponding portions in the total genotypic variance, 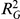 in an ideal F_2_ (under complete dominance at all segregating loci) or RI (DH) population.

F_2_ population		RI (DH) population	
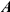	% of 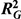	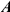	% of 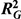
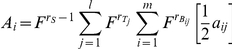	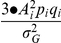	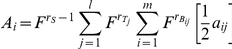	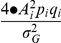
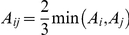	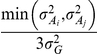	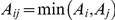	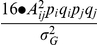
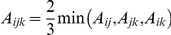	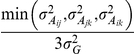	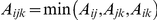	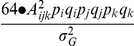
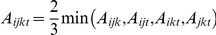	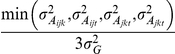	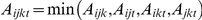	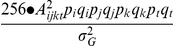

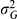
 is the expected genotypic variance for trait ***X*** in the population. In an F_2_, the QTL epistatic effects, 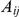, 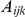, and 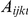 represent 1^st^, 2^nd^ and 3^rd^ order additive by additive epistasis parameters, respectively; % of 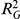 is the proportion of the total genotypic variation explained by 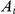, 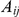, 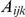, and 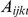, respectively. *p* and *q* are allelic frequencies of the two alleles at each locus involved, which is 0.5 in the ideal F_2_ and RI (DH) populations.

#### Theorem 3

In a RI (DH) population or under additivity for all loci, the estimated QTL epistatic effects, 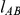, between the upstream loci and their downstream loci (type **I** epistasis) are equal to the estimated QTL main effects of the downstream loci, and the QTL epistatic effects between different loci within the same FGU (type **II** epistasis) are equal to their estimated QTL main effects. This is true for any high order epistasis (Table S4). However, for an F_2_ population (under complete dominance at all segregating loci), the QTL epistatic effects (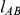) will equal two thirds of the estimated QTL main effects of the downstream loci, i.e. 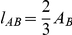, where *A* is the upstream locus and *B* is the downstream one for type **I** epistasis, and *A* and *B* are the different segregating loci within the same unit for type **II** epistasis (Table S4). Second-order epistasis involving alleles at 3 loci will equal two thirds of 1^st^ order epistasis, i.e. 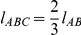, with progressively smaller contributions from higher-order epistasis. The coefficient, 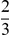 of the QTL epistatic effects, is the proportion of the heterozygote in the total functional genotypes in an F_2_ population (Tables 2, S4).

### Impact of epistasis on the classical biometrical genetics model

To better illustrate the two types of epistasis and compare the difference between **model (2)** and the ‘infinite’ model of classical quantitative genetics theory [31], we derived genetic expectations of the epistatic effects and predicted phenotypes (genotypic values) of multilocus genotypes involved in epistasis under scenario 3 (Tables S5, S6, S7). Interestingly, the QTL epistatic effects, 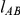, associated with the 4 digenic genotypes estimated by the classical quantitative genetics method, are inversely proportional to their expected frequencies in a population, even though the mean epistatic effect for any epistatic gene pair averaged across the 4 digenic genotypes is identical for both models. This implies that although **model (2)** and the classical biometrical genetics model are the same in predicting phenotypic values of multilocus genotypes in a RI (DH) population, the latter would overestimate the trait values of all multilocus genotypes in the presence of either type **I** or type **II** epistasis. Most importantly, the genetic expectations of multilocus genotypes involved in any type of epistasis in five of the 7 scenarios can be easily derived based on **model (2)**, providing the theoretical foundation for estimating the effects of segregating FGUs at each level of a signaling pathway. Also, the predicted patterns of phenotypic values associated with multilocus genotypes in the presence of epistasis by both models in a RI (DH) or F_2_ population are expected to result in greater trait variances among multilocus genotypes and thus increased power to detect epistasis as compared to detecting main-effect QTLs.

When the theory is extended to cover cases including two functional alleles with differentiated phenotypic values at any single loci in a signaling pathway of **model (2)**, the relationships between the mean pathway effects, 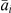, and the estimated QTL main effects, *A_j_* are as follows: 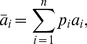
(4)

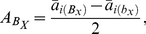
(5)

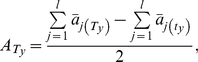
(6)

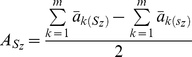
(7)where the coefficient, *p_i_*, is 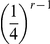 for an F_2_ population and 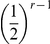 for a DH or RI population; 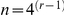 for an F_2_ population and 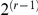 for a DH or RI population; 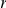 is the number of segregating loci in pathway *i*, 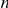 is the number of the specific group of genotypes with effect 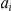, and 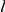 and 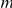 are the numbers of the pathways positively regulated by 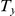 and 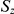, respectively. The capital and small letters of B, T and S in formula (5) - (7) represent two alleles of different trait values at each of the loci.

### Complementary epistasis may be of especially great importance

A major deviation from **Models (1)** and (**2**) is the presence of functionally differentiated alleles at different loci in a FGU. Table S8 shows a typical case, in which genes A and B represent 2 loci in the same FGU each with 2 functionally differentiated alleles, A_1_ and A_2_ at locus A, and B_1_ and B_2_ at locus B from the parents, P_1_ and P_2_. The parental-type digenic genotypes, A_1_A_1_B_1_B_1_ and A_2_A_2_B_2_B_2_ form two “co-adapted” di-allelic complexes with an equal pathway effect on trait ***X*** in the parents; but the recombinant-type digenic genotypes, A_1_A_1_B_2_B_2_ and A_2_A_2_B_1_B_1_ do not function well together and have no effect on trait ***X***. In this case, neither locus A nor locus B will be detected as a main-effect QTL (*A_A_* and *A_B_* = 0), but strong epistasis is detectable between loci A and B with an epistatic effect, *l_AB_* = 2.0, by classical QTL mapping methodology. The pathway effect for either 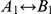 or 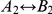 equals 2 x *l_AB_* = 4.0. In real situations, FGU 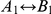 may not be equal to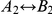.

### Genetic expectations of population parameters in response to selection

Figure S4 and Table S9 show the expected frequency shifts and population parameters in response to positive and negative selection under the 7 scenarios, which led us to two important results.

First, all segregating loci in the same FGU, whether in the regulatory (***S***, ***T***) or downstream (***B***) levels, have the same expected frequency shift in response to selection and their responses to positive and negative directional selections are generally asymmetric. Under positive selection, i.e. in the same direction as the pathway effect, all segregating loci in the pathway will show frequency shift in the direction of the pathway effect and following the order of ***S*** loci ≥***T*** loci ≥***B*** loci (particularly under additivity or in a RI/DH population). Under negative selection, i.e. in the opposite direction of the pathway effect, only null mutant alleles or repressors at upstream regulatory loci are responsive to selection and significant amounts of allelic diversity will remain at downstream loci.

Second, positive selection in the direction of the pathway effect will result in 2 types of weak positive linkage disequilibrium (LD), one between the upstream loci and their regulated downstream loci (type **I**, scenarios 3 and 4) and between different segregating loci within the same units in each level of the signaling pathway (scenarios 5–7). The number of any high order LDs can be predicted similarly based on the number of segregating loci and their functional relationships (Tables 1, S10), which can be estimated using Bennett's method [35]. For positively associated loci in the selected population, there are inclusive relationships between functional genotypes at the segregating upstream ***S*** or ***T*** loci and their downstream ***T*** or ***B*** loci in the same signaling pathway. The intensity of positive LD increases with selection intensity. Also, directional selection, either positive or negative in our simulation, results in many negative LDs between independent segregating loci in different FGUs of **model (2)**. This is artifactual, resulting from the functional redundancy of the downstream pathways - ***B*** units within **model (2)** (the preset stepwise trait increments are equal or proportional to the pathway effects of different ***B*** units in **model (2)**) (Fig. S4, Table S9). Once an FGU is included in the selected individuals, the remaining ones of equal effect will be excluded in these individuals, resulting in partial negative LDs between independent FGUs in the simulated populations.

### Detection of putative genetic networks underlying complex traits

Detecting and characterizing genetic networks underlying a complex trait involves determining the number, genetic relationships, and hierarchy of segregating FGUs (or loci) associated with the trait in a biparental population. Two general approaches are readily available - the quantitative genetics approach and the population genetics approach. The power to detect a genetic network is largely dependent on its complexity, which is determined largely by the number of segregating loci, 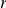, within each of the signaling pathways underlying the trait. Use of advanced BC or RI/DH populations can significantly increase power to detect a network for a complex trait when 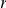
is large, by reducing the number of multilocus genotypes relative to an F_2_ population of maximum genetic complexity. In the following sections, we demonstrate both approaches by detecting putative genetic networks underlying plant height in a rice DH population and submergence tolerance (ST) in a set of selective introgression lines (ILs).

### Putative genetic network underlying plant height (PH) in rice

**Figure 2:**
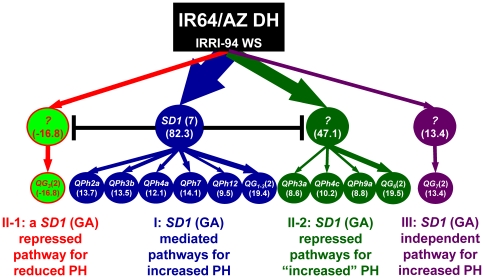
The putative genetic network underlying plant height (PH) of rice. It contains 3 major groups of functional genetic units (FGUs) or QTLs controlling rice PH. I - *SD1* (GA) mediated FGUs for increased PH; II-1 - a *SD1* (GA) repressed FGU for reduced PH; II-2 - *SD1* (GA) repressed FGUs with effects on PH of uncertain direction; and III – *SD1* (GA) independent FGU. The number under each FGU is its pathway effect estimated using the relevant QTL parameter of Table S11 and its genetic expectation (Tables S12, S13).

Using the 1994 wet-season data of the rice IR64/Azucena DH population [36], we identified the “Green Revolution” gene - *SD1* (*GA20ox-2*) [37], [38], 16 QTLs, and 11 pairwise epistatic interactions affecting PH at a threshold of P<0.0001 (Table S11). *GA20ox-2* encodes a key enzyme functioning in the biosynthetic pathway for gibberellic acids, GA_1_ and GA_4_, that play very important regulatory roles in rice growth and development [37]–[40]. A putative genetic network containing *SD1* and all 16 identified QTLs was constructed based on the theoretical expectations of their estimated main and epistatic effects (Fig. 2 and Tables S11, S12).

The network contained 3 major non-overlapping QTL groups. Group I was the *SD1* (GA) mediated pathways controlled by 6 independent FGUs, *QPh2a*, *QPh3b*, *QPh4a*, *QPh7b*, *QPh12* and *QG_1-3_* (*QPh8a* and *QPh9b*) which expressed (detectable) only in the presence of *SD1* (Fig. 2, Tables S11, S12, S13). Strong epistasis existed between *SD1* and the 6 FGUs. All these 6 downstream pathways had positive effects for increased height, ranging from 9.5 cm for *QPh12* to 19.4 cm for *QG_1-3_*. Together, the GA mediated pathways had a total estimated effect of 82.3 cm for increased PH (Fig. 2).

Group II was the *SD1* (GA) repressed pathways containing 5 FGUs of 2 types that expressed only in the *sd1* (mutant) background, but not in *SD1* (Fig. 2). Type 1 was *QG_3_* consisting of 2 interacting QTLs, *QPh3c* and *QPh7a*, with an estimated pathway effect of 16.8 cm for reduced PH. This is consistent with the reports on the presence of dominant semidwarf gene(s) in rice [41], [42]. Type 2 included 4 independent FGUs, *QPh3a*, *QPh4c*, *QPh9a* and *QG_6_* (*QPh1* and *QPh5a*) with estimated pathway effects of 8.6 cm, 10.2 cm, 8.8 cm and 19.5 cm, respectively. The pathway effect directions of *QPh3a*, *QPh4c*, and *QPh9a* could not be determined based on the known QTL information except *QG_6_*. Thus, the effect of the Green Revolution gene, *sd1*, on rice PH actually reflects the difference between the total effects of the GA mediated pathways versus the GA repressed pathways. The third group contains a single FGU (*QG_7_*) which was independent from *SD1*. This FGU contained 2 interacting QTLs, *QPh5b* and *QPh11*, with an estimated pathway effect of 13.4 cm for increased height.

### Putative genetic networks underlying submergence tolerance (ST) in rice detected by selective introgression

**Figure 3:**
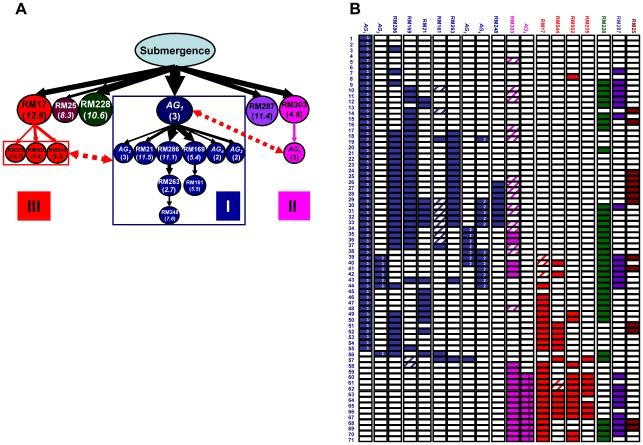
The putative genetic network underlying submergence tolerance (ST) of rice. (**A**) The multilocus structure consisting of 19 FGUs (14 loci and 5 *AGs*) in 3 major groups plus 3 independent loci identified in the 71 ST NPT/Khazar BC_3_ ILs (Table S14); (**B**) the graphic genotypes of the 71 ST NPT/Khazar BC_3_ ILs at the identified FGUs. The color boxes are homozygous donor (Khazar) alleles and patched boxes are the heterozygotes. An *AG* is a group of unlinked but perfectly associated loci identified in the selected ST ILs. Different orbits marked with different colors represent different FGUs either as single loci or as *AGs*. The number under each FGU is the source bin (marker) in the rice genome or the number of loci included in the *AG*.

*X^2^* tests at individual loci and the multilocus probability tests using genotypic data of 71 ST lines identified 19 FGUs, including 14 loci of excess introgression and 5 perfect association groups or AGs (groups of unlinked but perfectly associated loci in the selected ST ILs) based on a threshold of P<0.0001 (Table S14). LD analyses between the identified FGUs led us to the construction of a putative genetic network consisting of 3 major branches plus 3 independent loci (Fig. 3). Branch I had *AG_1_* (bins 5.5, 6.2 and 9.3) on the top connected with 9 largely independent and complementary downstream FGUs, including 6 loci near bins 5.4, 11.1, 11.5, 5.5, 2.7, and 7.6, *AG_2_* (bins 2.6, 5.1 and 5.3), *AG_3_* (bins 7.1 and 11.6) and *AG_4_* (bins 4.2 and 10.2). Together, this putative pathway was responsible for ST in 53 (74.6%) of the 71 ILs. The 3-locus FGU, *AG_1_* was detected independently in ST ILs from multiple populations and was always placed upstream of putative genetic networks for ST (unpublished data).

Branch II had bin 12.6 on the top connected with bins 8.2, 8.6 and 12.5 downstream. Branch III had bin 4.6 on the top associated with *AG_5_* (bins 2.5, 2.11 and 7.4) downstream. Three loci near bins 8.3, 10.6 and 11.4 formed 3 independent single-locus FGUs (Fig. 3). According to the theory developed above, branches (or FGU groups) I, II and III were most likely positively regulated pathways for improved ST, and the single locus FGU of high introgression at bin 10.6 was more likely a repressor for improved ST though it is difficult to determine the nature of the two other single-locus FGUs near bins 8.3 and 11.4 because of their relatively small effects (Fig. 3 and Table S14). Strong negative associations existed between the branch I loci and 3 downstream loci of branch II, and between *AG_1_* of branch I and downstream *AG_5_* of branch III, suggesting the possible presence of negative regulations between the putative pathways I and II, and between pathways I and III.

## Discussion

### Validity, predictions and deviations of the molecular-quantitative genetics theory

The theoretical framework of **models (1)** and **(2)** developed above is based on the notion that genetic variation of most complex traits is controlled by multiple signaling pathways, each involving many genes functioning in a strictly hierarchical manner as proven for many physiological and developmental traits in multicellular organisms, particularly their responses to internal and external perturbations [22], [23]. Thus, expression and development of any phenotypic traits controlled by gene networks start somehow in response to either internal or external stimuli, necessitating inclusion of two environmental elements, **E_1_** and **E_2_** in **model (1)** with **E_1_** (particularly, **E_S_**) being a key part of **model (1)**.

The principle of hierarchy and the existence of FGU are two important concepts in the theory. Hierarchy reflects the one-way FD of genes in downstream metabolic pathways on their upstream regulators, and the predicted relationships among function, number, effect size and diversity of genes in signaling pathways. FGU represents the most common type of functional relationships - referred to as “complementary epistasis” in many examples of classical genetics – and comprised of mutual function dependency among a group of related genes that affect phenotype(s) in a manner of a “house of cards” at each level of signaling pathways. Hierarchy and FGU in the theory suggest that epistasis results from two types of functional relationships between or among loci within a signaling pathway, each of which can be tracked to well known molecular mechanism(s). At the molecular level, type **I** epistasis can best be accounted for by protein-DNA interactions, protein-RNA and RNA-DNA interactions, while type **II** epistasis may primarily involve protein-protein interactions and enzyme cooperativity (Fig. S1, [34]).

Using **model (2)**, we demonstrated mathematical relationships between QTL main effects and epistatic effects and apparent correspondences between the quantitative genetics parameters and population genetic parameters of loci in signaling pathways, i.e. frequency shifts of loci and non-random association resulting from positive selection vs QTL main and epistatic effects identified in random segregating populations. One important assumption of the theory is that different ***T*** units act independently and their regulated downstream (***B***) units within a signaling pathway contribute to the trait(s) in the same direction, as in the case of the *SD1* (GA) mediated pathways for rice PH. This should be true in most cases because selection would not favor the repulsion situation that different ***T*** and ***B*** units within the same signaling pathway have opposite effects on the same traits, or a signaling pathway that is largely neutral with regard to its contribution to the trait or fitness. Thus, balancing selection would be more likely to act on different signaling pathways of opposite effects on a trait, rather than on different downstream pathways of opposite effects within a single signaling pathway.

Several important predictions from our theory can be used to test the generality of the model based on empirical results from typical QTL experiments. First, the theory predicts that many loci contributing to genetic variation of complex traits in natural populations are downstream (***B***) in pathways, and would be obscured in most QTL mapping studies in the presence of epistasis. This is because once a loss of function mutation occurs at any regulatory locus of a signaling pathway, mutations in its member genes and downstream FGUs may be relieved of selection pressure, except those having multiple functions.

Since unlinked genes of the same FGU may encode different enzymes or proteins of the same phenotypic effect(s), functional alleles of the same FGUs may show correlated responses to positive selection, resulting in non-random associations. However, the observed non-random associations between or among genetically independent FGUs for ST in rice were much stronger than the simulation results. For example, the largest pathway mediated by the 3-locus FGU, *AG_1_* was responsible for ST in 53 of the 71 ST ILs (Fig. 3). This 3-locus FGU was detected independently in ST ILs from multiple populations and always, when detected, placed in the upstream of putative genetic networks for ST (unpublished data), demonstrating the power and robustness of the population genetics approach in detecting genetic networks underlying complex traits. This type of strong non-random associations between or among unlinked loci was widely observed in ILs selected for ST, drought and salinity tolerances from large numbers of populations ([43]; unpublished data), suggesting some other mechanism(s), particularly **ER** in **model (1)**, in addition to strong selection, were responsible for the observed multilocus structure (unpublished data). Single FGUs, particularly the downstream ones, may be energetically the most efficient solutions to adaptive needs, but are genetically vulnerable both to mutations within their coding sequences and their upstream elements. Indeed, one can envision a ‘hierarchy of vulnerability’ with downstream FGUs being most vulnerable to loss of function. A tantalizing hypothesis for further study is that multicellular organisms have evolved multiple FGUs of similar or complementary function as a means to mitigate this vulnerability, which was proven to be the case in both our examples where each of the putative upstream FGUs are regulating multiple downstream FGUs of similar effects.

These predictions are consistent with both our examples and the observation that the complementary interaction was the predominant type of statistically detectable epistasis in rice QTL mapping studies [4]–[8], [44], and that most downstream loci tended to be detected as AGs in large selection experiments (unpublished data).

When extending the results from **model (2)** to **model (1)**, a third type of epistasis may exist, resulting from either antagonistic or synergistic relationships between loci in different signaling pathways, resulting in typical web-like networks detected in many genomewide gene expression studies [45]–[47]. Mathematically, both antagonistic and synergistic relationships cause statistical epistasis [48], as demonstrated in our examples including antagonistic epistasis (background effect) between *SD1* and its repressed PH QTLs (Fig. 2) and the strong negative LDs between loci of two different lineages (putative pathways) in the ST network, and with empirical examples of antagonistic epistasis between loci in the GA and ABA signaling pathways [23] and synergistic epistasis between loci of the GA and ethylene pathways [49], [50].

We find that responses to divergent selection for a complex trait controlled by signaling pathways are generally asymmetrical in a segregating population unless only one locus in a pathway is segregating. This is because function (phenotype) can be much more easily altered by disabling a FGU than restoring it by recombination. Thus, we offer an alternative explanation of the asymmetrical responses observed in many artificial selection experiments [51]–[56].

Both epistasis and genotype-by-environment interaction play a central role in maintaining genetic variation for complex traits in populations, even under strong directional selection. This is because a similar phenotype or fitness under a given environment can be achieved by various combinations of signaling pathways with opposite effects. Indeed, multicellular organisms may adapt to fluctuating environments through multiple alternative signaling pathways of similar but not identical functions. This provides an excellent explanation for the observed correlation between environmental heterogeneity and genetic diversity in plant populations [57], [58]. Results from large selection experiments for several abiotic stress tolerances in rice (unpublished data) provide strong evidence in support of this prediction. In other words, differential expression of regulatory genes, particularly those functioning at level **S**, may be largely responsible for the observed GE interaction of complex traits, as observed in previous QTL studies [36], [59].

Our model suggests that both loss of function and “co-adapted” gene complexes formed by multiple alleles with differentiated functions (effects) should be frequent types of allelic diversity at loci that contribute to the genetic variation of complex traits in populations. Observations from numerous studies [4]–[8], [44], [60] appear to lend strong support to this inference, and resequencing studies have recently begun to reveal surprisingly high frequencies of apparently-crippling mutations in natural plant populations [61].

Finally, our model predicts that heterosis for most complex traits would arise primarily from complementarity between dominant or partially dominant regulatory genes and additive downstream ones, and inbreeding depression is due primarily to the breakdown of functioning FGUs by recombination. The theory further predicts that a high level of heterosis may be found in crosses between ecotypes adapted to highly-differentiated environments because they tend to carry different signaling pathways related to fitness and its components. These predictions are consistent with numerous empirical observations in both plants and animals and have been proven to be true in two large series of experiments in rice [5]–[10], [16], [36], [44]. In other words, it seems reasonable to assume complete or partial dominance as an important characteristic for regulatory genes in signaling pathways. Nevertheless, this assumption remains to be tested in future experiments.

Extending the above results to include multiple signaling pathways and the random noise of E_2_ of **model (1)** where trait heterosis is averaged across all involved segregating pathways, the mixed mode of gene action appears to fit more closely to real situations of most complex traits. This is because trait heterosis under complete dominance or additivity for all loci (Table S3 and Fig. S3) contradicts the commonly observed importance of additive gene actions for most quantitative traits and the observed variation in the levels and directions of heterosis. In the latter case, ∼50% of the negative trait heterosis under additivity does not appear to fit the observed heterosis for most complex traits, particularly fitness and its components.

### Deviations of the two models from real ones

Both **models (1)** and **(2)** are very much simplified relative to current knowledge of signaling pathways. At the molecular level, there are complex webs of relationships between genes/pathways within a signaling pathway, including multiple transcriptional factor binding sites [62], multiple phosphorylations [62], and genetic “redundancy” from gene duplications in both copy number and function, each of which can result in deviations from **models (1)** and **(2)**. More complicated relationships of FD in signaling pathways such as multiple TF binding sites and phosphorylations may result in the “redundant” branch pattern in genetic networks [63], as seen in our example of the rice ST genetic network. Genetic redundancy is expected to generate the “distributed” branching pattern, which is included in **model (2)** (different ***T*** units and different ***B*** units within each ***T*** unit of are actually ‘redundant’ with their effects on trait ***X***) and well demonstrated in the *SD1* example. These deviations are expected to result in more complex branching patterns and more layers of genetic networks as long as these relationships fall into one of the two major types with regard to their effects on specific phenotype(s) defined in this paper. Consistent with our expectation, selection experiments from more than 80 backcross populations revealed that the redundant branching pattern was prevalent in rice genetic networks for drought tolerance (DT) and ST (unpublished data). Thus, the general “distributed” pattern of genetic networks underlying DT and ST in rice suggests a significant level of downstream genetic redundancy, consistent with our current understanding of most plant abiotic stress signaling pathways [64].

### Detection and verification

With the theory developed above, additional modeling efforts are needed to combine this model with stochastic features of linear statistical and landscape models for parameter estimation, genetic network construction and genotype-phenotype prediction in breeding populations. As demonstrated in our first example, the current statistical methodology and modeling tools for QTL analyses and genotype-phenotype predictions [24], [25]; [29], [30], [65] remain valid, because the estimated QTL parameters can be readily converted to the pathway effects based on their genetic expectations developed in this study. It is important to point out that all detected putative genetic networks should be verified, particularly when identified in single environments. In our first example, the rice PH network (and the FGUs) was consistently detected across multiple diverse environments, providing interesting insights into the nature of Green Revolution (unpublished data). However, a major limitation of the quantitative genetics approach was noted, i.e. the direction of a pathway (FGU) effect can not be determined if it is not involved in any epistasis, as seen in most of FGUs in the GA repressed pathways. In our second example, similar rice ST networks were identified in ILs from more than 20 populations, indicating the robustness of the method (unpublished data).

There are several ways to verify the identified FGUs and their relationships within genetic networks. Random reciprocal introgression lines derived from the same parents are ideal for both quantitative and population genetics approaches. A second way is to use progeny testing. As demonstrated in our two examples, selected lines of extreme phenotype from a segregating population represent unique multilocus genotypes at the identified FGUs based on which the genetic network for the selected trait was constructed. Backcrossing each line to the recurrent parent can create a segregating population to test and verify the identified genetic network using either population or quantitative genetics. In addition, lines created for verification are also suitable for high-throughput -omic analyses and extensive phenotypic evaluation to identify genes underlying the genetic networks by an integrated approach [43].

## Materials and Methods

### The materials and method of the quantitative genetics approach

In case study 1, the materials used are the well-known IR64/Azucena doubled haploid (DH) population of 126 rice lines with the plant height (PH) data obtained in the 1995 dry-season at IRRI and genotypic data of 176 RFLP markers [36]. Three steps were taken to detect the putative genetic network underlying PH using the quantitative genetics approach: (1) to identify main-effect and digenic epistatic QTLs affecting PH in the population using the classical QTL mapping approach [24]; (2) to determine the relationships between and among the identified QTLs based on their epistasis and the magnitudes of their QTL main effects; and (3) to estimate the pathway effects of independent QTL groups based on the genetic expectations of the multilocus genotypes of interacting QTLs demonstrated in Table S6.

### The materials and method of population genetics approach

In case study 2, the materials included 71 rice introgression lines (ILs) with significantly improved ST selected from 1900 BC_3_F_2_ plants from a cross between a new plant type (NPT) line and Khazar. NPT is a tropical japonica line developed at IRRI and used as the recipient. Khazar is a *japonica* landrace from Iran and used as the donor. The initial cross was made in 1998 and the F_1_ plants were backcrossed to the recipient to obtain BC_1_F_1_ seeds, from which 25 random BC_1_F_1_ plants were each backcrossed to the RP to produce 25 BC_2_F_1_ lines. Then, 1–3 random plants in each of the BC_2_F_1_ lines were further backcrossed to the RP to produce 30–75 BC_3_F_1_ lines. The resultant BC_3_F_1_ lines were planted in the field and allowed to self to produce BC_3_F_2_ seeds. Seeds from all BC_3_F_1_ plants were bulk-harvested as a single BC_3_F_2_ population. In the 2001–2002 dryseason, 1900 BC_3_F_2_ plants of the NPT/Khazar cross were subjected to 2-week complete submergence in the deep-water pond of the IRRI experimental farm, resulting in 71 survival plants (unpublished data). ST of the survival plants were confirmed in the progeny testing under 2-week complete submergence during the following 2002 wet-season. Then, a total of 625 SSR markers across the rice genome were used to screen the polymorphisms between the RP and ST donor, from which 159 polymorphic SSR markers were used to genotype the ST ILs (unpublished data).

According to the classical population genetics theory and our computer simulation, 3 steps were taken to detect the putative genetic network underlying ST in the ILs. First, we performed two types of statistical tests to identify FGUs associated with ST. We used standard *X^2^* tests to detect donor alleles at individual loci across the genome that deviated significantly in both allelic and genotypic frequencies from the expectations and multilocus probability tests to detect individual association groups (AGs) in the selected ST ILs using the formula 
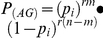
. Here, an AG is defined as a group of 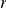 (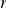
≥2) perfectly associated but genetically unlinked loci of equal introgression in the ST ILs, where 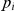 is the expected frequency of the donor introgression in a BC_3_F_2_ population, 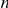 is the number of ILs, 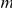 is the number of ILs that have co-introgression of the donor alleles, and 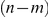 is the number of ILs having no introgression at the *r* unlinked loci in the AG. Thus, (*P_i_*)*^m^* is the probability of *m* ILs having co-introgression of the donor alleles and (1 - *P_i_*)*^n^* ^-*m*^ is the probability of (*n*-*m*) ILs having no introgression at *r* unlinked loci. For *r* loci (*r*≥2) in an AG, there will be *r*(*r*−1)/2 independent pairwise associations between the *r* loci. The threshold to claim a significant case was *P*≤0.0001 for individual cases. Second, we performed pairwise gametic LD analyses to determine non-random associations between individual FGUs identified in the first step using the standard approach [66]. Third, we constructed the putative genetic network or the multilocus structure containing all identified FGUs based on the principle of hierarchy in 2 steps: (1) all FGUs detected in the ILs were divided based on the LD results into major groups such that different FGUs within each group were all significantly and positively associated with one another (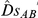 = 1.0), and FGUs in different groups were either independent, or negatively associated; and (2) all FGUs within each group were connected, forming multiple layers, according to their progressively reduced introgression and inclusive relationships. The underlying assumption for the network construction is that all FGUs in a network are unlinked, which was true in our case because all redundant loci due to linkage associated with each of the identified FGUs were removed.

## Supporting Information

Table S1 Nature of allelic diversity at 26 cloned QTLs.
(0.07 MB DOC)

Table S2 Quantitative genetics presentation of multilocus zygote genotypes and their corresponding phenotypic effect, *a_ij_* (assuming complete dominance) of three unlinked segregating loci of a single functional genetic unit (FGU) in an F_2_ population with two alleles at each of the loci, one functional allele (the capital letter) and the other nonfunctional mutant (the small letter).
(0.05 MB DOC)

Table S3 The expected F_1_ values, mid-parental heterosis (*H*_MP_), and population parameters of an ideal F_2_ or RI (DH) population derived from a biparental cross under different gene actions and the seven scenarios in Table 1 and model (2) of Figure 1B.
(0.05 MB DOC)

Table S4 Expected QTL and population parameters for nine functional genetic units (FGUs), including one *S* unit, two *T* units and six *B* units of a signaling pathway defined in model (2) (Figure 1B) under the seven scenarios defined in Table 1, regarding the number of segregating loci in each of these FGUs in populations derived from a cross between two inbred parents, P_1_ and P_2_.
(0.18 MB DOC)

Table S5 Comparison between the estimated epistatic effects of four digenic genotypes for each epistatic loci pair under scenario 3 (Figure 1B and Table 1) and their phenotypic values assigned based on model (2) in an F_2_ or RI (DH) population.
(0.06 MB DOC)

Table S6 The genetic expectations and phenotypic values of digenic genotypes in trait *X* based on model (2) and the classic quantitative genetics model under scenario 3 (Figure 1B and Table 1) in a RI or DH population.
(0.10 MB DOC)

Table S7 The genetic expectations and phenotypic values of digenic genotypes in trait *X* predicted based on model (2) and the classic quantitative genetic model under scenario 3 (Figure 1B and Table 1) in an F_2_ (complete dominance) population.
(0.10 MB DOC)

Table S8 Complementary epistasis affecting trait *X* in the presence of functionally differentiated alleles at two segregating loci, A and B, in an FGU in an RI (DH) population derived from parents, P_1_ and P_2_.
(0.05 MB DOC)

Table S9 Expected population parameters, *μ* (mean) and *σ* ^2^_G_ (variance), of segregating loci in a signaling pathway of model (2) resulting from positive and negative selection under the seven scenarios of biparental populations defined in Table 1.
(0.28 MB DOC)

Table S10 Nonrandom associations, measured by the normalized gametic linkage disequilibrium statistics (LD'), between loci segregating in a signaling pathway of model (2) resulting from positive and negative selection for increased trait values under the seven scenarios of an RI (or DH) populations defined in Table 1.
(0.26 MB DOC)

Table S11 Genetic parameters of 16 QTLs in seven groups (*QG*) affecting plant heights identified in the IR64/Azucena DH population evaluated in 1994 wet season at IRRI [36].
(0.07 MB DOC)

Table S12 Inferred effects on plant height (cm) of the *SD1* mediated downstream pathways (*QG_1-3_*, *QPh8a* and *QPh9b*) based on the theoretical expectations and observed plant heights (in cm) of the tri-locus genotypes at the corresponding QTLs.
(0.06 MB DOC)

Table S13 Inferred effects of the *SD1* mediated QTL groups (*QG1* expect for *QG_1-3_*), *QG3*, *QG6* and *QG7* on plant height based on the theoretical expectations and observed plant heights of the multilocus genotypes at the corresponding loci.
(0.15 MB DOC)

Table S14 Identification of 19 functional genetic units (FGUs) affecting submergence tolerance (ST) by *χ* ^2^ tests (single loci) and multi-locus probability tests in 71 ST introgression lines selected from 1900 BC_3_F_2_ plants derived from the cross between NPT (recurrent parent) and Khazar (donor).
(0.08 MB DOC)

Figure S1 Hypothetical molecular mechanisms involved in a positively regulated signaling pathway affecting trait *X*, in which a signal from a specific environmental factor, ES, is perceived by one or more receptor proteins either directly or through an smRNA, each encoded by a single gene, forming a single signal transduction (S) unit. The transduction unit then induces the expression of six transcriptional factor genes forming two separate protein complexes, T_1_ and T_2_, units. T_1_ and T_2_ then each regulate a set of downstream genes *B_111_*, *B_112_*, *B_113_*, etc.; encoding enzymes En_111_, En_112_, En_113_, etc. or *B_211_*, *B_212_*, etc. encoding En_211_, En_212_, etc. that function in downstream pathway B_11_ or B_21_, resulting in metabolites M_11_ or M_21_, which has phenotypic effect *a_11_* or *a_21_* on trait *X*. Sub_111_, Sub_112_, and Sub_113_ are biochemical substrates of enzymes En_111_, En_112_, En_113_ encoded by genes *B_111_*, *B_112_*, *B_113_*, respectively.
(1.49 MB TIF)

Figure S2 Expected frequency distributions of the phenotypic values of trait *X* in an F_2_ and a recombinant inbred line population segregating at different numbers of loci in a single signaling pathway of model (2) under the seven scenarios defined and Table 1 and Figure 1B.
(1.13 MB TIF)

Figure S3 The expected mid-parental trait heterosis (*H*_MP_) under three types of gene actions under scenarios 3–7 of Table 1 regarding the type and number of segregating loci in a signaling pathway defined in Figure 1B. In the mixed gene action, all segregating loci at regulatory (*S* and *T*) levels are completely dominant, and all loci at the downstream level *B* act additively. *r* and *n* are the numbers of segregating loci and possible distributions of the segregating loci in the parents.
(1.64 MB TIF)

Figure S4 The expected cumulated frequency shifts of functional alleles in response to positive and negative selection under the seven scenarios defined in Table 1 (A) under complete dominance at all segregating loci in an F_2_ population, (B) under mixed gene action (complete dominance for the regulatory *S* and *T* loci and additivity for the downstream *B* loci) in an F_2_ population, (C) complete additivity in an F_2_ population, and (D) RI or DH population. In the steps of selection, 1, 2, …, 8 represent the selection trait thresholds of ≥4.0 or ≤4.0, …, ≥32.0 or ≤32.0 for positive or negative selection defined in Table S9.
(0.51 MB PDF)
