# Supplementary material for: Dissecting Genetic Networks Underlying Complex Phenotypes: The Theoretical Framework
Source: PLoS One. 2011 Jan 20;6(1):e14541. doi: 10.1371/journal.pone.0014541 (PMC3024316; doi:10.1371/journal.pone.0014541)
Supplement: Table S1 — Nature of allelic diversity at 26 cloned QTLs. (0.07 MB DOC) [file pone.0014541.s001.doc]

**Supplementary Tables**

**Table S1** Nature of allelic diversity at 26 cloned QTLs

| Species | Trait | Gene | Allelic diversity ^1^ | Regulator | Reference |
| --- | --- | --- | --- | --- | --- |
| Rice | Salt tolerance | *SKC1* | LOF | No | 1 |
| Rice | Grain width | *GW2* | LOF | Yes | 2 |
| Rice | Heading date | *HD6* | LOF | Yes | 3 |
| Rice | Grain length and weight | *GS3* | LOF | Yes | 4 |
| Rice | Grain yield/flowering time etc | *Ghd7* | LOF | Yes | 5 |
| Rice | Grain yield | *IPA1 /OsSPL14* | DT | RNAi | 6 |
| Rice | Grain yield/panicle angle | *DEP1* | GOF | Yes | 7 |
| Rice | Grain yield | *Gn1a /OsCKX2* | LOF | Yes | 8 |
| Rice | Tiller angle | *PROG1* | LOF | Yes | 9 |
| Rice | Plant height | *sd1* | LOF | Yes | 10 |
| Rice | Grain width | *qSW5* | LOF | ? | 11 |
| Rice | Blast disease resistance | *Pi21* | LOF | Yes | 12 |
| Rice | Heading date | *HD1* | LOF | Yes | 13 |
| Rice | Heading date | *Hd3a* | DT | Yes | 14 |
| Rice | Submergence tolerance | *Sub1A* | LOF | Yes | 15 |
| Wheat | Grain protein/zinc/iron content | *Gpc-B1* | LOF | Yes | 16 |
| Wheat | Gibberellin response modulator | *Rht-1* | GOF | Yes | 17 |
| Maize | Oil content and composition | *qHO6* | FD/DT | No | 18 |
| Tomato | Sugar yield | *Brix9-2-5* | LOF | No | 19 |
| Tomato | Iron | *chloronerva* | LOF | No | 20 |
| Arabidopsis | Flowering time | *EDI/Cry2* | LOF | Yes | 21 |
| Arabidopsis | Salt tolerance/ABA sensitivity | *RAS1* | LOF | Yes | 22 |
| Mouse | Macrophage iron burden | *Mon1a* | GOF | No | 23 |
| Mouse | Immobile behavior | *Usp46* | DT | Yes | 24 |
| Human | Autosomal recessive retinitis pigmentosa | *RP25/EYS* | LOF | No | 25 |
| Human | Cardiac arrhythmias | *KVLQT1* | DT | No | 26 |

FD = functional differences, LOF = loss of functional mutant. GOF = gain of functional mutant. DT = differential transcripts.

**References:**

1. Ren ZH, Gao JP, Li LG, Cai XL, Huang W, et al. (2005) A rice quantitative trait locus for salt tolerance encodes a sodium transporter. Nature Genetics 37: 1141-1146.
2. Song XJ, Huang W, Shi M, Zhu MZ, Lin HX (2007) A QTL for rice grain width and weight encodes a previously unknown RING-type E3 ubiquitin ligase. Nature genetics 39: 623-630.
3. [Takahashi](http://www.pnas.org/search?author1=Yuji+Takahashi&sortspec=date&submit=Submit) Y, [Shomura](http://www.pnas.org/search?author1=Ayahiko+Shomura&sortspec=date&submit=Submit) A, [Sasaki](http://www.pnas.org/search?author1=Takuji+Sasaki&sortspec=date&submit=Submit) T, [Yano](http://www.pnas.org/search?author1=Masahiro+Yano&sortspec=date&submit=Submit) M (2001) *Hd6*, a rice quantitative trait locus involved in photoperiod sensitivity, encodes the α subunit of protein kinase CK2. Proc. Natl. Acad. Sci. USA 98: 7922–7927.
4. Fan CC, Xing YZ, Mao HL, Lu TT, Han B, et al. (2006) *GS3*, a major QTL for grain length and weight and minor QTL for grain width and thickness in rice, encodes a putative transmembrane protein. Theor Appl Genet 112: 1164–1171.
5. Xue WY, Xing YZ, Weng XY, Zhao Y, Tang WJ, et al. (2008) Natural variation in *Ghd7* is an important regulator of heading date and yield potential in rice. Nature genetics 40: 761-767.
6. Jiao YQ, Wang YH, Xue DW, Wang J, Yan MX, et al. (2010) Regulation of *OsSPL14* by OsmiR156 defines ideal plant architecture in rice. Nature Genetics 42: 541-544.
7. Huang XZ, Qian Q, Liu ZB, Sun HY, He SY, et al. (2009) Natural variation at the *DEP1* locus enhances grain yield in rice. Nature Genetics 41: 494-497.
8. Ashikari M, Sakakibara H, Lin SY, Yamamoto T, Takashi T, et al. (2005) Cytokinin oxidase regulates rice grain production. Science 309: 741-745.
9. Tan LB, Li XR, Liu FX, Sun XY, Li CG, et al. (2008) Control of a key transition from prostrate to erect growth in rice domestication. Nature Genetics 40: 1360-1364.
10. [Sasaki A](http://www.ncbi.nlm.nih.gov/pubmed?term="Sasaki A"%5BAuthor%5D), [Ashikari M](http://www.ncbi.nlm.nih.gov/pubmed?term="Ashikari M"%5BAuthor%5D), [Ueguchi-Tanaka M](http://www.ncbi.nlm.nih.gov/pubmed?term="Ueguchi-Tanaka M"%5BAuthor%5D), [Itoh H](http://www.ncbi.nlm.nih.gov/pubmed?term="Itoh H"%5BAuthor%5D), [Nishimura A](http://www.ncbi.nlm.nih.gov/pubmed?term="Nishimura A"%5BAuthor%5D), et al. (2002) A mutant gibberellin-synthesis gene in rice. Nature 416: 701-702.
11. Shomura A, Izawa T, Ebana K, Ebitani T, Kanegae H, et al. (2008) Deletion in a gene associated with grain size increased yields during rice domestication. Nature Genetics 40: 1023-1028.
12. Fukuoka S, Saka N, Koga H, Ono K, Shimizu T, et al. (2009) Loss of function of a proline-containing protein confers durable disease resistance in rice. Science 325 : 998-1001.
13. Yano M, Katayose Y, Ashikari M, Yamanouchi U, Monna L, et al. (2000) *Hd1*, a Major photoperiod sensitivity quantitative trait locus in rice, is closely related to the Arabidopsis flowering time gene *CONSTANS*. The Plant Cell 12: 2473-2483.
14. Kojima S, Takahashi Y, Kobayashi Y, Monna L, Sasaki T, et al. (2002) *Hd3a*, a rice ortholog of the *Arabidopsis* *FT* gene, promotes transition to flowering downstream of *Hd1* under short-day conditions. Plant Cell Physiol. 43: 1096-1105.
15. Xu KN, Xu X, Fukao T, Canlas P, Maghirang-Rodriguez R, et al. (2006) *Sub1A* is an ethylene-response-factor-like gene that confers submergence tolerance to rice. Nature 442: 705-708.
16. Uauy C, Distelfeld A, Fahima T, Blechl A, Dubcovsky Jorge (2006) A NAC gene regulating senescence improves grain protein, zinc, and iron content in wheat. Science 314: 1298-1301.
17. Peng JR, Richards DE, Hartley NM, Murphy GP, Devos KM, et al. (1999) ‘Green revolution’ genes encode mutant gibberellins response modulators. Nature 400: 256-261.
18. Zheng PZ, Allen WB, Roesler K, Williams ME, Zhang SR, et al. (2008) A phenylalanine in DGAT is a key determinant of oil content and composition in maize. Nature Genetics 40: 367-372.
19. Fridman E, Carrari F, Liu YS, Fernie AR, Zamir D (2004) Zooming in on a quantitative trait for tomato yield using interspecific introgressions. Science 305: 1786-1789.
20. Ling HQ, Koch G, Baumlein H, Ganal MW (1999) Map-based cloning of *chloronerva*, a gene involved in iron uptake of higher plants encoding nicotianamine synthase. Proc. Natl. Acad. Sci. USA 96: 7098-7103.
21. El-Din El-Assal S, Alonso-Blanco C, Peeters AJ, Raz V, Koornneef M (2001) A QTL for flowering time in *Arabidopsis* reveals a novel allele of *CRY2*. Nature Genetics 29: 435-440.
22. Ren ZH, Zheng ZM, Chinnusamy V, Zhu JH, Cui XP, et al. (2010) RAS1, a quantitative trait locus for salt tolerance and ABA sensitivity in *Arabidopsis*. Proc. Natl. Acad. Sci. USA 107: 5669-5674.
23. Wang FD, Paradkar PN, Custodio AO, McVey Ward D, Fleming MD, et al. (2007) Genetic variation in *Mon1a* affects protein trafficking and modifies macrophage iron loading in mice. Nature Genetics 39: 1025-1032.
24. Tomida S, Mamiya T, Sakamaki H, Miura M, Aosaki T, et al. (2009) *Usp46* is a quantitative trait gene regulating mouse immobile behavior in the tail suspension and forced swimming tests. Nature Genetics 41: 688-695.
25. Abd El-Aziz MM, Barragan I, O’Driscoll CA, Goodstadt L, Prigmore E, et al. (2008) *EYS*, encoding an ortholog of *Drosophila* spacemaker, is mutated in autosomal recessive retinitis pigmentosa. Nature Genetics 40: 1285-1287.
26. Wang Q, Curran ME, Splawski I, Burn TC, Millholland JM, et al. (1996) Positional cloning of a novel potassium channel gene: *KVLQT1* mutations cause cardiac arrhythmias. Nature Genetics 12: 17-23.
