# Supplementary material for: Dissecting Genetic Networks Underlying Complex Phenotypes: The Theoretical Framework
Source: PLoS One. 2011 Jan 20;6(1):e14541. doi: 10.1371/journal.pone.0014541 (PMC3024316; doi:10.1371/journal.pone.0014541)
Supplement: Table S2 — Quantitative genetics presentation of multilocus zygote genotypes and their corresponding phenotypic effect, aij (assuming complete dominance) of three unlinked segregating loci of a single functional genetic unit (FGU) in an F2 population with two alleles at each of the loci, one functional allele (the capital letter) and the other nonfunctional mutant (the small letter). (0.05 MB DOC) [file pone.0014541.s002.doc]

**Table S2.** Quantitative genetics presentation of multilocus zygote genotypes and their corresponding phenotypic effect, *a_ij_* (assuming complete dominance) of 3 unlinked segregating loci of a single functional genetic unit (FGU) in an F_2_ population with two alleles at each of the loci - one functional allele (the capital letter) and the other nonfunctional mutant (the small letter)

| Female |  | Male gamete | | | | | | | |
| --- | --- | --- | --- | --- | --- | --- | --- | --- | --- |
| gamete |  | ABC | AbC | ABc | Abc | aBC | aBc | abC | abc |
| ABC | Genotype | A-B-C- | A-B-C- | A-B-C- | A-B-C- | A-B-C- | A-B-C- | A-B-C- | A-B-C- |
|  | Phenotype | *a_ij_* | *a_ij_* | *a_ij_* | *a_ij_* | *a_ij_* | *a_ij_* | *a_ij_* | *a_ij_* |
| AbC | Genotype | A-B-C- | A-bbC- | A-B-C- | A-bbC- | A-B-C- | A-B-C- | A-bbC- | A-bbC- |
|  | Phenotype | *a_ij_* | 0 | *a_ij_* | 0 | *a_ij_* | *a_ij_* | 0 | 0 |
| ABc | Genotype | A-B-C- | A-B-C- | A-B-cc | A-B-cc | A-B-C- | A-B-cc | A-B-C- | A-B-cc |
|  | Phenotype | *a_ij_* | *a_ij_* | 0 | 0 | *a_ij_* | 0 | *a_ij_* | 0 |
| Abc | Genotype | A-B-C- | A-bbC- | A-B-cc | A-bbC- | A-B-C- | A-B-cc | A-bbC- | A-bbcc |
|  | Phenotype | *a_ij_* | 0 | 0 | 0 | *a_ij_* | 0 | 0 | 0 |
| aBC | Genotype | A-B-C- | A-B-C- | A-B-C- | A-B-C- | aaB-C- | aaB-C- | aaB-C- | aaB-C- |
|  | Phenotype | *a_ij_* | *a_ij_* | *a_ij_* | *a_ij_* | 0 | 0 | 0 | 0 |
| aBc | Genotype | A-B-C- | A-B-C- | A-B-cc | A-B-cc | aaB-C- | aaB-cc | aabbC- | aaB-cc |
|  | Phenotype | *a_ij_* | *a_ij_* | 0 | 0 | 0 | 0 | 0 | 0 |
| abC | Genotype | A-B-C- | A-bbC- | A-B-C- | A-bbC- | aaB-C- | aaB-C- | aabbC- | aabbC- |
|  | Phenotype | *a_ij_* | 0 | *a_ij_* | 0 | 0 | 0 | 0 | 0 |
| abc | Genotype | A-B-C- | A-bbC- | A-B-cc | A-bbcc | aaB-C- | aa-B-cc | aabbC- | aabbcc |
|  | Phenotype | *a_ij_* | 0 | 0 | 0 | 0 | 0 | 0 | 0 |
