# Supplementary material for: Dissecting Genetic Networks Underlying Complex Phenotypes: The Theoretical Framework
Source: PLoS One. 2011 Jan 20;6(1):e14541. doi: 10.1371/journal.pone.0014541 (PMC3024316; doi:10.1371/journal.pone.0014541)
Supplement: Table S3 — The expected F1 values, mid-parental heterosis (H MP), and population parameters of an ideal F2 or RI (DH) population derived from a biparental cross under different gene actions and the seven scenarios in Table 1 and model (2) of Figure 1B. (0.05 MB DOC) [file pone.0014541.s003.doc]

**Table S3.** The expected F_1_ values, mid-parental heterosis (*H*_MP_), and population parameters of an ideal F_2_ or RI (DH) population derived from a biparental cross under different gene actions and 7 scenarios in Table 1 and **model (2)** of Fig. 1B

|  |  | **Complete dominance** | | | | | **Additivity** | | | | | **Mixed ^2^** | | | | | **DH/RI** | |
| --- | --- | --- | --- | --- | --- | --- | --- | --- | --- | --- | --- | --- | --- | --- | --- | --- | --- | --- |
|  |  | **F_1_** | ***H_MP_*** | | **F_2_** | | **F_1_** | ***H_MP_*** | | **F_2_** | | **F_1_** | ***H_MP_*** | | **F_2_** | |
| **Scenario** | **N ^1^** |  | $\overline{H}\pm SD$ | **Range** | $\mu$ | $\sigma_{G}^{2}$ |  | $\overline{H}\pm SD$ | **Range** | $\mu$ | $\sigma_{G}^{2}$ |  | $\overline{H}\pm SD$ | **Range** | $\mu$ | $\sigma_{G}^{2}$ | $\mu$ | $\sigma_{G}^{2}$ |
| **1** | 2 | 28.0 | 14.0±0.0 | 14.0-14.0 | 21.0 | 75.0 | 14.0 | 0.0±0.0 | 0.0-0.0 | 14.0 | 50.0 | 28.0 | 14.0±0.0 | 14.0-14.0 | 21.0 | 75.0 | 14.0 | 100.0 |
| **2** | 8 | 32.0 | 10.0±0.0 | 10.0-10.0 | 27.0 | 21.0 | 22.0 | 0.0±0.0 | 0.0-0.0 | 22.0 | 14.0 | 22.0 | 0.0±0.0 | 0.0-0.0 | 22.0 | 14.0 | 22.0 | 28.0 |
| **3** | 42 | 32.0 | 21.3±2.8 | 16.0-26.0 | 20.3 | 84.2 | 11.0 | 0.3±2.8 | -5.0-5.0 | 11.0 | 35.8 | 22.0 | 11.3±2.8 | 6.0-16.0 | 16.5 | 56.3 | 11.0 | 75.0 |
| **4** | 64 | 32.0 | 26.5±4.4 | 16.0-32.0 | 15.2 | 140.0 | 5.5 | 0.0±4.4 | -10.5-5.5 | 5.5 | 28.5 | 22.0 | 16.5±4.4 | 6.0-22.0 | 12.4 | 93.2 | 5.5 | 67.8 |
| **5** | 42 | 32.0 | 29.5±4.1 | 16.0-32.0 | 11.8 | 140.6 | 3.0 | 0.5±4.1 | -13.0-3.0 | 3.0 | 15.6 | 32.0 | 29.5±4.1 | 16.0-32.0 | 11.8 | 140.6 | 3.0 | 47.0 |
| **6** | 64 | 32.0 | 21.0±1.6 | 16.0-22.0 | 19.2 | 77.0 | 11.0 | 0.0±1.6 | -5.0-1.0 | 11.0 | 32.6 | 22.0 | 11.0±1.6 | 6.0-12.0 | 16.5 | 49.6 | 11.0 | 67.5 |
| **7** | 163 | 32.0 | 19.7±1.7 | 14.0-22.0 | 20.0 | 47.7 | 12.5 | 0.2±1.7 | -5.5-2.5 | 12.5 | 15.5 | 15.0 | 2.7±1.7 | -3.0-5.0 | 13.8 | 19.5 | 12.5 | 33.8 |
| **Average** | 55 | 31.4 | 20.3±6.2 |  | 19.2 | 83.6 | 11.3 | 0.1±0.2 |  | 11.3 | 27.4 | 23.3 | 12.1±9.0 |  | 16.3 | 64.0 | 11.3 | 59.9 |

^1^ N is the total number of possible mutilocus parental genotypes of different phenotypes; ^2^ The specification of simulation includes 3 types of gene action: complete dominance, complete additivity and mixed gene actions (complete dominance at ***S*** and ***T*** loci and additivity at ***B*** loci).
