# Supplementary material for: Dissecting Genetic Networks Underlying Complex Phenotypes: The Theoretical Framework
Source: PLoS One. 2011 Jan 20;6(1):e14541. doi: 10.1371/journal.pone.0014541 (PMC3024316; doi:10.1371/journal.pone.0014541)
Supplement: Table S4 — Expected QTL and population parameters for nine functional genetic units (FGUs), including one S unit, two T units and six B units of a signaling pathway defined in model (2) (Figure 1B) under the seven scenarios defined in Table 1, regarding the number of segregating loci in each of these FGUs in populations derived from a cross between two inbred parents, P1 and P2. (0.18 MB DOC) [file pone.0014541.s004.doc]

**Table S4.** Expected QTL and population parameters for 9 functional genetic units (FGUs), including 1 ***S*** unit, 2 ***T*** units and 6 ***B*** units of a signaling pathway defined in **model (2)** (Fig. 1B) under 7 scenarios (Table 1) in populations derived from a cross between 2 inbred parents, P1 and P2

|  |  | F_2_ | | | | | | RI or DH | |
| --- | --- | --- | --- | --- | --- | --- | --- | --- | --- |
|  | Simulated | Dominance ^1^ | | DA | | Additivity | |
| Scenario | parameters ^2^ | $A$ | $R_{G}^{2}$(%) | $A$ | $R_{G}^{2}$(%) | $A$ | $R_{G}^{2}$(%) | $A$ | $R_{G}^{2}$(%) |
| **1** | ***T_1_*** | 8.0 | 64.00 | 8.0 | 64.00 | 8.0 | 64.00 | 8.0 | 64.0 |
|  | ***T_2_*** | 6.0 | 36.00 | 6.0 | 36.00 | 6.0 | 36.00 | 6.0 | 36.0 |
| **2** | ***B_11_*** | 2.0 | 14.30 | 2.0 | 14.30 | 2.0 | 14.30 | 2.0 | 14.3 |
|  | ***B_13_*** | 2.0 | 14.30 | 2.0 | 14.30 | 2.0 | 14.30 | 2.0 | 14.3 |
|  | ***B_21_*** | 4.0 | 57.10 | 4.0 | 57.10 | 4.0 | 57.10 | 4.0 | 57.1 |
|  | ***B_23_*** | 2.0 | 14.30 | 2.0 | 14.30 | 2.0 | 14.30 | 2.0 | 14.3 |
| **3** | ***T_1_*** | 7.0 | 43.65 | 6.0 | 48.00 | 6.0 | 50.35 | 6.0 | 48.0 |
|  | ***B_11_*** | 1.5 | 1.95 | 1.5 | 2.00 | 1.0 | 1.40 | 1.0 | 1.3 |
|  | ***B_13_*** | 1.5 | 1.95 | 1.5 | 2.00 | 1.0 | 1.40 | 1.0 | 1.3 |
|  | ***T_1_-B_11_*** | 1.0 | 0.67 | 1.0 | 0.67 | 1.0 | 0.70 | 1.0 | 1.3 |
|  | ***T_1_-B_13_*** | 1.0 | 0.67 | 1.0 | 0.67 | 1.0 | 0.70 | 1.0 | 1.3 |
|  | ***T_2_*** | 6.5 | 37.65 | 5.0 | 33.30 | 5.0 | 34.97 | 5.0 | 33.3 |
|  | ***B_21_*** | 3.0 | 7.95 | 3.0 | 8.00 | 2.0 | 5.59 | 2.0 | 5.3 |
|  | ***B_23_*** | 1.5 | 1.95 | 1.5 | 2.00 | 1.0 | 1.40 | 1.0 | 1.3 |
|  | ***T_2_-B_21_*** | 2.0 | 2.67 | 2.0 | 2.67 | 2.0 | 2.80 | 2.0 | 5.3 |
|  | ***T_2_-B_23_*** | 1.0 | 0.67 | 1.0 | 0.67 | 1.0 | 0.70 | 1.0 | 1.3 |
| **4** | ***S*** | 10.125 | 54.91 | 8.25 | 54.75 | 5.5 | 53.01 | 5.50 | 44.6 |
|  | ***T_1_*** | 5.25 | 14.76 | 4.5 | 16.29 | 3.0 | 15.77 | 3.00 | 13.3 |
|  | ***B_11_*** | 1.125 | 0.68 | 1.125 | 0.68 | 0.5 | 0.44 | 0.50 | 0.4 |
|  | ***B_13_*** | 1.125 | 0.68 | 1.125 | 0.68 | 0.5 | 0.44 | 0.50 | 0.4 |
|  | ***T_2_*** | 4.875 | 12.73 | 3.75 | 11.31 | 2.5 | 10.95 | 2.50 | 9.2 |
|  | ***B_21_*** | 2.25 | 2.71 | 2.25 | 2.71 | 1.0 | 1.75 | 1.00 | 1.5 |
|  | ***B_23_*** | 1.125 | 0.68 | 1.125 | 0.68 | 0.5 | 0.44 | 0.50 | 0.4 |
|  | ***S-T_1_*** | 3.5 | 4.92 | 3.0 | 5.43 | 3.0 | 7.89 | 3.00 | 13.3 |
|  | ***S-B_11_*** | 0.75 | 0.23 | 0.75 | 0.23 | 0.5 | 0.22 | 0.50 | 0.4 |
|  | ***S-B_13_*** | 0.75 | 0.23 | 0.75 | 0.23 | 0.5 | 0.22 | 0.50 | 0.4 |
|  | ***S-T_1_-B_11_*** | 0.5 | 0.08 | 0.5 | 0.08 | 0.5 | 0.11 | 0.50 | 0.4 |
|  | ***S-T_1_-B_13_*** | 0.5 | 0.08 | 0.5 | 0.08 | 0.5 | 0.11 | 0.50 | 0.4 |
|  | ***T_1_-B_11_*** | 0.75 | 0.23 | 0.75 | 0.23 | 0.5 | 0.22 | 0.50 | 0.4 |
|  | ***T_1_-B_13_*** | 0.75 | 0.23 | 0.75 | 0.23 | 0.5 | 0.22 | 0.50 | 0.4 |
|  | ***S-T_2_*** | 3.25 | 4.24 | 2.5 | 3.77 | 2.5 | 5.48 | 2.50 | 9.2 |
|  | ***S-B_21_*** | 1.5 | 0.90 | 1.5 | 0.90 | 1.0 | 0.88 | 1.00 | 1.5 |
|  | ***S-B_23_*** | 0.75 | 0.23 | 0.75 | 0.23 | 0.5 | 0.22 | 0.50 | 0.4 |
|  | ***T_2_-B_21_*** | 1.5 | 0.90 | 1.5 | 0.90 | 1.0 | 0.88 | 1.00 | 1.5 |
|  | ***T_2_-B_23_*** | 0.75 | 0.23 | 0.75 | 0.23 | 0.5 | 0.22 | 0.50 | 0.4 |
|  | ***S-T_2_-B_21_*** | 1.0 | 0.30 | 1.0 | 0.30 | 1.0 | 0.44 | 1.00 | 1.5 |
|  | ***S-T_2_-B_23_*** | 0.5 | 0.08 | 0.5 | 0.08 | 0.5 | 0.11 | 0.50 | 0.4 |
| **5** | ***S*** | 7.875 | 33.09 | 7.875 | 33.09 | 3.0 | 28.92 | 3.0 | 19.1 |
|  | ***T_11_*** | 4.5 | 10.80 | 4.5 | 10.80 | 2.0 | 12.85 | 2.0 | 8.5 |
|  | ***T_12_*** | 4.5 | 10.80 | 4.5 | 10.80 | 2.0 | 12.85 | 2.0 | 8.5 |
|  | ***T_21_*** | 3.375 | 6.08 | 3.375 | 6.08 | 1.0 | 3.21 | 1.0 | 2.1 |
|  | ***T_22_*** | 3.375 | 6.08 | 3.375 | 6.08 | 1.0 | 3.21 | 1.0 | 2.1 |
|  | ***T_23_*** | 3.375 | 6.08 | 3.375 | 6.08 | 1.0 | 3.21 | 1.0 | 2.1 |
|  | ***S-T_11_*** | 3.0 | 3.60 | 3.0 | 3.60 | 2.0 | 6.43 | 2.0 | 8.5 |
|  | ***S-T_12_*** | 3.0 | 3.60 | 3.0 | 3.60 | 2.0 | 6.43 | 2.0 | 8.5 |
|  | ***T_11_-T_12_*** | 3.0 | 3.60 | 3.0 | 3.60 | 2.0 | 6.43 | 2.0 | 8.5 |
|  | ***S-T_11_-T_12_*** | 2.0 | 1.20 | 2.0 | 1.20 | 2.0 | 3.21 | 2.0 | 8.5 |
|  | ***S-T_21_*** | 2.25 | 2.03 | 2.25 | 2.03 | 1.0 | 1.61 | 1.0 | 2.1 |
|  | ***S-T_22_*** | 2.25 | 2.03 | 2.25 | 2.03 | 1.0 | 1.61 | 1.0 | 2.1 |
|  | ***S-T_23_*** | 2.25 | 2.03 | 2.25 | 2.03 | 1.0 | 1.61 | 1.0 | 2.1 |
|  | ***T_21_-T_22_*** | 2.25 | 2.03 | 2.25 | 2.03 | 1.0 | 1.61 | 1.0 | 2.1 |
|  | ***T_21_-T_23_*** | 2.25 | 2.03 | 2.25 | 2.03 | 1.0 | 1.61 | 1.0 | 2.1 |
|  | ***T_22_-T_23_*** | 2.25 | 2.03 | 2.25 | 2.03 | 1.0 | 1.61 | 1.0 | 2.1 |
|  | ***S-T_21_-T_22_*** | 1.5 | 0.68 | 1.5 | 0.68 | 1.0 | 0.80 | 1.0 | 2.1 |
|  | ***S-T_21_-T_23_*** | 1.5 | 0.68 | 1.5 | 0.68 | 1.0 | 0.80 | 1.0 | 2.1 |
|  | ***S-T_22_-T_23_*** | 1.5 | 0.68 | 1.5 | 0.68 | 1.0 | 0.80 | 1.0 | 2.1 |
|  | ***T_21_-T_22_-T_23_*** | 1.5 | 0.68 | 1.5 | 0.68 | 1.0 | 0.80 | 1.0 | 2.1 |
|  | ***S-T_21_-T_22_-T_23_*** | 1.0 | 0.23 | 1.0 | 0.23 | 1.0 | 0.40 | 1.0 | 2.1 |
| **6** | ***T_1_*** | 7.125 | 49.45 | 6.5 | 63.89 | 6.5 | 64.78 | 6.5 | 62.6 |
|  | ***B_111_*** | 1.125 | 1.23 | 0.75 | 0.57 | 0.5 | 0.38 | 0.5 | 0.4 |
|  | ***B_112_*** | 1.125 | 1.23 | 0.75 | 0.57 | 0.5 | 0.38 | 0.5 | 0.4 |
|  | ***T_1_-B_111_*** | 0.75 | 0.41 | 0.5 | 0.19 | 0.5 | 0.19 | 0.5 | 0.4 |
|  | ***T_1_-B_112_*** | 0.75 | 0.41 | 0.5 | 0.19 | 0.5 | 0.19 | 0.5 | 0.4 |
|  | ***B_111_-B_112_*** | 0.75 | 0.41 | 0.75 | 0.28 | 0.5 | 0.19 | 0.5 | 0.4 |
|  | ***T_1_-B_111_-B_112_*** | 0.5 | 0.14 | 0.5 | 0.09 | 0.5 | 0.10 | 0.5 | 0.4 |
|  | ***T_2_*** | 5.6875 | 31.51 | 4.5 | 30.62 | 4.5 | 31.00 | 4.5 | 30.0 |
|  | ***B_211_*** | 1.6875 | 2.77 | 0.75 | 0.57 | 0.5 | 0.38 | 0.5 | 0.4 |
|  | ***B_212_*** | 1.6875 | 2.77 | 0.75 | 0.57 | 0.5 | 0.38 | 0.5 | 0.4 |
|  | ***B_213_*** | 1.6875 | 2.77 | 0.75 | 0.57 | 0.5 | 0.38 | 0.5 | 0.4 |
|  | ***T_2_-B_211_*** | 1.125 | 0.92 | 0.5 | 0.19 | 0.5 | 0.19 | 0.5 | 0.4 |
|  | ***T_2_-B_212_*** | 1.125 | 0.92 | 0.5 | 0.19 | 0.5 | 0.19 | 0.5 | 0.4 |
|  | ***T_2_-B_213_*** | 1.125 | 0.92 | 0.5 | 0.19 | 0.5 | 0.19 | 0.5 | 0.4 |
|  | ***T_2_-B_211_-B_212_*** | 0.75 | 0.31 | 0.5 | 0.09 | 0.5 | 0.10 | 0.5 | 0.4 |
|  | ***T_2_-B_211_-B_213_*** | 0.75 | 0.31 | 0.5 | 0.09 | 0.5 | 0.10 | 0.5 | 0.4 |
|  | ***T_2_-B_212_-B_213_*** | 0.75 | 0.31 | 0.5 | 0.09 | 0.5 | 0.10 | 0.5 | 0.4 |
|  | ***B_211_-B_212_*** | 1.125 | 0.92 | 0.75 | 0.28 | 0.5 | 0.19 | 0.5 | 0.4 |
|  | ***B_211_-B_213_*** | 1.125 | 0.92 | 0.75 | 0.28 | 0.5 | 0.19 | 0.5 | 0.4 |
|  | ***B_212_-B_213_*** | 1.125 | 0.92 | 0.75 | 0.28 | 0.5 | 0.19 | 0.5 | 0.4 |
|  | ***B_211_-B_212_-B_213_*** | 0.75 | 0.31 | 0.75 | 0.14 | 0.5 | 0.10 | 0.5 | 0.4 |
|  | ***T_2_-B_211_-B_212_-B_213_*** | 0.5 | 0.10 | 0.5 | 0.05 | 0.5 | 0.05 | 0.5 | 0.4 |
| **7** | ***B_12_*** | 4.0 | 25.16 | 4.0 | 41.09 | 4.0 | 51.56 | 4.0 | 47.3 |
|  | ***B_13_*** | 2.0 | 6.29 | 2.0 | 10.27 | 2.0 | 12.89 | 2.0 | 11.8 |
|  | ***T_2_*** | 4.6875 | 34.56 | 2.5 | 24.08 | 2.5 | 20.14 | 2.5 | 18.5 |
|  | ***B_211_*** | 1.6875 | 4.48 | 0.75 | 1.44 | 0.5 | 0.81 | 0.5 | 0.7 |
|  | ***B_212_*** | 1.6875 | 4.48 | 0.75 | 1.44 | 0.5 | 0.81 | 0.5 | 0.7 |
|  | ***B_213_*** | 1.6875 | 4.48 | 0.75 | 1.44 | 0.5 | 0.81 | 0.5 | 0.7 |
|  | ***B_22_*** | 1.5 | 3.54 | 1.5 | 5.78 | 1.0 | 3.22 | 1.0 | 3.0 |
|  | ***B_23_*** | 1.5 | 3.54 | 1.5 | 5.78 | 1.0 | 3.22 | 1.0 | 3.0 |
|  | ***T_2_-B_211_*** | 1.125 | 1.49 | 0.5 | 0.48 | 0.5 | 0.40 | 0.5 | 0.7 |
|  | ***T_2_-B_212_*** | 1.125 | 1.49 | 0.5 | 0.48 | 0.5 | 0.40 | 0.5 | 0.7 |
|  | ***T_2_-B_213_*** | 1.125 | 1.49 | 0.5 | 0.48 | 0.5 | 0.40 | 0.5 | 0.7 |
|  | ***B_211_-B_212_*** | 1.125 | 1.49 | 0.75 | 0.72 | 0.5 | 0.40 | 0.5 | 0.7 |
|  | ***B_211_-B_213_*** | 1.125 | 1.49 | 0.75 | 0.72 | 0.5 | 0.40 | 0.5 | 0.7 |
|  | ***B_212_-B_213_*** | 1.125 | 1.49 | 0.75 | 0.72 | 0.5 | 0.40 | 0.5 | 0.7 |
|  | ***B_211_-B_212_-B_213_*** | 0.75 | 0.50 | 0.75 | 0.36 | 0.5 | 0.20 | 0.5 | 0.7 |
|  | ***T_2_-B_211_-B_212_*** | 0.75 | 0.50 | 0.5 | 0.24 | 0.5 | 0.20 | 0.5 | 0.7 |
|  | ***T_2_-B_211_-B_213_*** | 0.75 | 0.50 | 0.5 | 0.24 | 0.5 | 0.20 | 0.5 | 0.7 |
|  | ***T_2_-B_212_-B_213_*** | 0.75 | 0.50 | 0.5 | 0.24 | 0.5 | 0.20 | 0.5 | 0.7 |
|  | ***T_2_-B_211_-B_212_-B_213_*** | 0.5 | 0.17 | 0.5 | 0.12 | 0.5 | 0.10 | 0.5 | 0.7 |
|  | ***T_2_-B_22_*** | 1.0 | 1.18 | 1.0 | 1.93 | 1.0 | 1.61 | 1.0 | 3.0 |
|  | ***T_2_-B_23_*** | 1.0 | 1.18 | 1.0 | 1.93 | 1.0 | 1.61 | 1.0 | 3.0 |

1. QTL parameters include 2 major types: $A$ representing QTL main and epistatic effects between or among loci in the same unit(s), or between/among alleles at the upstream loci and loci in the downstream unit(s), assuming complete dominance at loci at all levels in the system.
2. All estimated QTL epistatic effects between loci in different ***T*** units or between loci in different ***B*** units are zero, and thus not listed in the table. All possible but non-listed epistasis between loci in different ***B*** units equals to zero.
