# Supplementary material for: Dissecting Genetic Networks Underlying Complex Phenotypes: The Theoretical Framework
Source: PLoS One. 2011 Jan 20;6(1):e14541. doi: 10.1371/journal.pone.0014541 (PMC3024316; doi:10.1371/journal.pone.0014541)
Supplement: Table S5 — Comparison between the estimated epistatic effects of four digenic genotypes for each epistatic loci pair under scenario 3 (Figure 1B and Table 1) and their phenotypic values assigned based on model (2) in an F2 or RI (DH) population. (0.06 MB DOC) [file pone.0014541.s005.doc]

**Table S5.** Comparison between the estimated epistatic effects of 4 digenic genotypes for each epistatic loci pair under scenario 3 (Fig. 1B and Table 1) and their phenotypic values assigned based on **model (2)** in an F_2_ or RI (DH) population

|  |  | **F_2_** | | | | | **RI or DH** | | | | |
| --- | --- | --- | --- | --- | --- | --- | --- | --- | --- | --- | --- |
|  |  | **9** | **3** | **3** | **1** |  | **1** | **1** | **1** | **1** |  |
| *A* vs *B* | **Model** | ***A-B-*** | ***A-bb*** | ***aaB-*** | ***aabb*** | **Mean** | ***AABB*** | ***AAbb*** | ***aaBB*** | ***aabb*** | **Mean** |
| *T_1_* vs *B_11_* | **Model (2)** | 4.0 | 0 | 0 | 0 | 1.0 | 4.0 | 0 | 0 | 0 | 1.0 |
|  | Classic | 0.25 | -0.75 | -0.75 | 2.25 | 1.0 | 1.0 | -1.0 | -1.0 | 1.0 | 1.0 |
| *T_1_* vs *B_13_* | **Model (2)** | 4.0 | 0 | 0 | 0 | 1.0 | 4.0 | 0 | 0 | 0 | 1.0 |
|  | Classic | 0.25 | -0.75 | -0.75 | 2.25 | 1.0 | 1.0 | -1.0 | -1.0 | 1.0 | 1.0 |
| *T_2_* vs *B_21_* | **Model (2)** | 8.0 | 0 | 0 | 0 | 2.0 | 8.0 | 0 | 0 | 0 | 2.0 |
|  | Classic | 0.5 | -1.5 | -1.5 | 4.5 | 2.0 | 2.0 | -2.0 | -2.0 | 2.0 | 2.0 |
| *T_2_* vs *B_23_* | **Model (2)** | 4.0 | 0 | 0 | 0 | 1.0 | 4.0 | 0 | 0 | 0 | 1.0 |
|  | Classic | 0.25 | -0.75 | -0.75 | 2.25 | 1.0 | 1.0 | -1.0 | -1.0 | 1.0 | 1.0 |
