# Supplementary material for: Dissecting Genetic Networks Underlying Complex Phenotypes: The Theoretical Framework
Source: PLoS One. 2011 Jan 20;6(1):e14541. doi: 10.1371/journal.pone.0014541 (PMC3024316; doi:10.1371/journal.pone.0014541)
Supplement: Table S6 — The genetic expectations and phenotypic values of digenic genotypes in trait X based on model (2) and the classic quantitative genetics model under scenario 3 (Figure 1B and Table 1) in a RI or DH population. (0.10 MB DOC) [file pone.0014541.s006.doc]

**Table S6.** The genetic expectations and phenotypic values of digenic genotypes in trait *X* based on **model (2)** and the classic quantitative genetics model under scenario 3 (Fig. 1B and Table 1) in a RI or DH population

| Epistasis |  | **Digenic genotypes in an RI (DH) population** | | | |
| --- | --- | --- | --- | --- | --- |
|  | **Model** | **A-B-** | **A-bb** | **aaB-** | **aabb** |
|  | **Model (2)** | 19.0 | 15.0 | 5.0 | 5.0 |
| (***T_1_*** vs ***B_11_***) |  | $A_{B_{11}}+A_{B_{12}}+\frac{1}{2}A_{B_{13}}+C_{1}$ | $A_{B_{12}}+\frac{1}{2}A_{B_{13}}+C_{1}$ | $C_{1}$ | $C_{1}$ |
|  | Classic | 19.0 | 15.0 | 5.0 | 5.0 |
|  |  | $\mu+2A_{A}+2A_{B}+l_{AB}$ | $\mu+2A_{A}-2A_{B}-l_{AB}$ | $\mu-2A_{A}+2A_{B}-l_{AB}$ | $\mu-2A_{A}-2A_{B}+l_{AB}$ |
| (***T_1_*** vs ***B_13_***) | **Model (2)** | 19.0 | 15.0 | 5.0 | 5.0 |
|  |  | $A_{B_{11}}+A_{B_{13}}+\frac{1}{2}A_{B{}_{12}}+C_{1}$ | $A_{B_{12}}+\frac{1}{2}A_{B_{11}}+C_{1}$ | $C_{1}$ | $C_{1}$ |
|  | Classic | 19.0 | 15.0 | 5.0 | 5.0 |
| (***T_2_*** vs ***B_21_***) | **Model (2)** | 20.0 | 12.0 | 6.0 | 6.0 |
|  |  | $A_{B_{21}}+A_{B_{22}}+\frac{1}{2}A_{B_{23}}+C_{2}$ | $A_{B_{22}}+\frac{1}{2}A_{B_{23}}+C_{2}$ | $C_{2}$ | $C_{2}$ |
|  | Classic | 20.0 | 12.0 | 6.0 | 6.0 |
| (***T_2_*** vs ***B_23_***) | **Model (2)** | 18.0 | 14.0 | 6.0 | 6.0 |
|  |  | $A_{B_{23}}+A_{B_{22}}+\frac{1}{2}A_{B_{21}}+C_{2}$ | $A_{B_{22}}+\frac{1}{2}A_{B_{21}}+C_{2}$ | $C_{2}$ | $C_{2}$ |
|  | Classic | 18.0 | 14.0 | 6.0 | 6.0 |

^1^ , and .
