# Supplementary material for: Dissecting Genetic Networks Underlying Complex Phenotypes: The Theoretical Framework
Source: PLoS One. 2011 Jan 20;6(1):e14541. doi: 10.1371/journal.pone.0014541 (PMC3024316; doi:10.1371/journal.pone.0014541)
Supplement: Table S7 — The genetic expectations and phenotypic values of digenic genotypes in trait X predicted based on model (2) and the classic quantitative genetic model under scenario 3 (Figure 1B and Table 1) in an F2 (complete dominance) population. (0.10 MB DOC) [file pone.0014541.s007.doc]

**Table S7.** The genetic expectations and phenotypic values of digenic genotypes in trait *X* predicted based on **model (2)** and the classic quantitative genetic model under scenario 3 (Fig. 1B and Table 1) in an F_2_ (complete dominance) population

|  |  | **Digenic genotypes in an F_2_ population** | | | |
| --- | --- | --- | --- | --- | --- |
| Epistasis | **Model** | **A-B-** | **A-bb** | **aaB-** | **aabb** |
| (T_1_ vs B_11_) | **Model (2)** | 24.75 | 20.75 | 9.75 | 9.75 |
|  |  | $A_{B_{11}}+A_{B_{12}}+\frac{3}{4}A_{B_{13}}+C_{5}$ | $A_{B_{12}}+\frac{3}{4}A_{B_{13}}+C_{5}$ | $C_{5}$ | $C_{5}$ |
|  | Classic | 29.75 | 24.75 | 13.75 | 12.75 |
|  |  | $\mu+2A_{A}+2A_{B}+l_{AB}$ | $\mu+2A_{A}-2A_{B}-l_{AB}$ | $\mu-2A_{A}+2A_{B}-l_{AB}$ | $\mu-2A_{A}-2A_{B}+l_{AB}$ |
| (T_1_ vs B_13_) | **Model (2)** | 24.75 | 20.75 | 9.75 | 9.75 |
|  |  | $A_{B_{13}}+A_{B_{12}}+\frac{3}{4}A_{B_{11}}+C_{5}$ | $A_{B_{12}}+\frac{3}{4}A_{B_{11}}+C_{5}$ | $C_{5}$ | $C_{5}$ |
|  | Classic | 29.75 | 24.75 | 13.75 | 12.75 |
| (T_2_ vs B_21_) | **Model (2)** | 25.5 | 17.5 | 10.5 | 10.5 |
|  |  | $A_{B_{21}}+A_{B_{22}}+\frac{3}{4}A_{B_{23}}+C_{6}$ | $A_{B_{22}}+\frac{3}{4}A_{B_{23}}+C_{6}$ | $C_{6}$ | $C_{6}$ |
|  | Classic | 31.75 | 21.75 | 14.75 | 12.75 |
| (T_2_ vs B_23_) | **Model (2)** | 24.5 | 20.5 | 10.5 | 10.5 |
|  |  | $A_{B_{23}}+A_{B_{22}}+\frac{3}{4}A_{B_{21}}+C_{6}$ | $A_{B_{22}}+\frac{3}{4}A_{B_{21}}+C_{6}$ | $C_{6}$ | $C_{6}$ |
|  | Classic | 29.25 | 24.25 | 14.25 | 13.25 |

^1^ $C_{5}=\frac{9}{16}A_{B_{21}}+\frac{3}{4}A_{B_{22}}+\frac{9}{16}A_{B_{23}}$ and $C_{6}=\frac{9}{16}A_{B_{11}}+\frac{3}{4}A_{B_{12}}+\frac{9}{16}A_{B_{13}}$.
