# Supplementary material for: Dissecting Genetic Networks Underlying Complex Phenotypes: The Theoretical Framework
Source: PLoS One. 2011 Jan 20;6(1):e14541. doi: 10.1371/journal.pone.0014541 (PMC3024316; doi:10.1371/journal.pone.0014541)
Supplement: Table S8 — Complementary epistasis affecting trait X in the presence of functionally differentiated alleles at two segregating loci, A and B, in an FGU in an RI (DH) population derived from parents, P1 and P2. (0.05 MB DOC) [file pone.0014541.s008.doc]

**Table S8.** Complementary epistasis affecting trait *X* in the presence of functionally differentiated alleles at two segregating loci, A and B in a FGU in a RI (DH) population derived from parents, P_1_ and P_2_

|  | Digenic genotypes | | | | QTL effects | | Pathway effects | |
| --- | --- | --- | --- | --- | --- | --- | --- | --- |
|  | A_1_A_1_B_1_B_1_ | A_1_A_1_B_2_B_2_ | A_2_A_2_B_1_B_1_ | A_2_A_2_B_2_B_2_ | $A_{A_{1}}$or $A_{B_{1}}$ | $l_{A_{1}B_{1}}$or $l_{A_{2}B_{2}}$ | $A_{1}\leftrightarrow B_{1}$ | $A_{2}\leftrightarrow B_{2}$ |
| Trait values | 4.0 | 0 | 0 | 4.0 | 0 | 2.0 | 4.0 | 4.0 |
