# Supplementary material for: Dissecting Genetic Networks Underlying Complex Phenotypes: The Theoretical Framework
Source: PLoS One. 2011 Jan 20;6(1):e14541. doi: 10.1371/journal.pone.0014541 (PMC3024316; doi:10.1371/journal.pone.0014541)
Supplement: Table S9 — Expected population parameters, μ (mean) and σ 2 G (variance), of segregating loci in a signaling pathway of model (2) resulting from positive and negative selection under the seven scenarios of biparental populations defined in Table 1. (0.28 MB DOC) [file pone.0014541.s009.doc]

**Table S9.** Expected population parameters, $\mu$(mean) and $\sigma_{G}^{2}$(variance), of segregating loci in a signaling pathway of model (2) resulting from positive and negative selection under the 7 scenarios of biparental populations defined in Table 1

|  |  | |  | |  |  | |  | |  | F_2_ | |  | |  |  | |  | |  | RI (DH) | | |
| --- | --- | --- | --- | --- | --- | --- | --- | --- | --- | --- | --- | --- | --- | --- | --- | --- | --- | --- | --- | --- | --- | --- | --- |
|  | Selection | | | | | Complete dominance | | | | | Mixed | | | | | Additivity | | | | | Additivity | | |
| **Scenario** |  | Step | | TT ^1^ | | S.I. ^2^ | $\mu$ | | $\sigma_{G}^{2}$ | | S.I. | $\mu$ | | $\sigma_{G}^{2}$ | | S.I. | $\mu$ | | $\sigma_{G}^{2}$ | | S.I. | $\mu$ | $\sigma_{G}^{2}$ |
|  |  | 8 | | ≥32 | | 0.000 | 0.0 | | 0.0 | | 0.000 | 0.0 | | 0.0 | | 0.000 | 0.0 | | 0.0 | | 0.000 | 0.0 | 0.0 |
|  |  | 7 | | ≥28 | | 0.560 | 28.0 | | 0.0 | | 0.560 | 28.0 | | 0.0 | | 0.060 | 28.0 | | 0.0 | | 0.250 | 28.0 | 0.0 |
|  | PS | 6 | | ≥24 | | 0.560 | 28.0 | | 0.0 | | 0.560 | 28.0 | | 0.0 | | 0.060 | 28.0 | | 0.0 | | 0.500 | 22.0 | 72.0 |
|  |  | 5 | | ≥20 | | 0.560 | 28.0 | | 0.0 | | 0.560 | 28.0 | | 0.0 | | 0.310 | 22.4 | | 8.6 | | 0.500 | 22.0 | 72.0 |
|  |  | 4 | | ≥16 | | 0.750 | 25.0 | | 27.0 | | 0.750 | 25.0 | | 27.0 | | 0.380 | 21.3 | | 12.9 | | 0.500 | 22.0 | 72.0 |
|  |  | 3 | | ≥12 | | 0.940 | 22.4 | | 48.6 | | 0.940 | 22.4 | | 48.6 | | 0.690 | 17.8 | | 22.1 | | 0.750 | 18.7 | 69.3 |
|  |  | 2 | | ≥8 | | 0.940 | 22.4 | | 48.6 | | 0.940 | 22.4 | | 48.6 | | 0.810 | 16.3 | | 31.3 | | 0.750 | 18.7 | 69.3 |
|  |  | 1 | | ≥4 | | 0.940 | 22.4 | | 48.6 | | 0.940 | 22.4 | | 48.6 | | 0.940 | 14.9 | | 39.4 | | 0.750 | 18.7 | 69.3 |
| **1** |  | 0 | |  | | 1.000 | 21.0 | | 75.0 | | 1.000 | 21.0 | | 75.0 | | 1.000 | 14.0 | | 50.0 | | 1.000 | 14.0 | 133.0 |
|  |  | 1 | | <32 | | 1.000 | 21.0 | | 75.0 | | 1.000 | 21.0 | | 75.0 | | 1.000 | 14.0 | | 50.0 | | 1.000 | 14.0 | 133.0 |
|  |  | 2 | | <28 | | 0.440 | 12.0 | | 27.4 | | 0.440 | 12.0 | | 27.4 | | 0.940 | 13.1 | | 39.4 | | 0.750 | 9.3 | 69.3 |
|  | NS | 3 | | <24 | | 0.440 | 12.0 | | 27.4 | | 0.440 | 12.0 | | 27.4 | | 0.940 | 13.1 | | 39.4 | | 0.750 | 9.3 | 69.3 |
|  |  | 4 | | <20 | | 0.440 | 12.0 | | 27.4 | | 0.440 | 12.0 | | 27.4 | | 0.690 | 10.2 | | 22.1 | | 0.750 | 9.3 | 69.3 |
|  |  | 5 | | <16 | | 0.250 | 9.0 | | 27.0 | | 0.250 | 9.0 | | 27.0 | | 0.630 | 9.6 | | 20.6 | | 0.500 | 6.0 | 72.0 |
|  |  | 6 | | <12 | | 0.060 | 0.0 | | 0.0 | | 0.060 | 0.0 | | 0.0 | | 0.310 | 5.6 | | 8.6 | | 0.250 | 0.0 | 0.0 |
|  |  | 7 | | <8 | | 0.060 | 0.0 | | 0.0 | | 0.060 | 0.0 | | 0.0 | | 0.190 | 4.0 | | 8.0 | | 0.250 | 0.0 | 0.0 |
|  |  | 8 | | <4 | | 0.060 | 0.0 | | 0.0 | | 0.060 | 0.0 | | 0.0 | | 0.060 | 0.0 | | 0.0 | | 0.250 | 0.0 | 0.0 |
|  |  |  | |  | |  |  | |  | |  |  | |  | |  |  | |  | |  |  |  |
|  |  | 8 | | ≥32 | | 0.316 | 32.0 | | 0.0 | | 0.004 | 32.0 | | 0.0 | | 0.004 | 32.0 | | 0.0 | | 0.060 | 32.0 | 0.0 |
|  |  | 7 | | ≥28 | | 0.633 | 30.0 | | 4.0 | | 0.094 | 28.7 | | 1.2 | | 0.094 | 28.7 | | 1.2 | | 0.250 | 29.0 | 4.0 |
|  | PS | 6 | | ≥24 | | 0.844 | 28.5 | | 9.8 | | 0.398 | 25.7 | | 3.7 | | 0.398 | 25.7 | | 3.7 | | 0.500 | 26.5 | 8.9 |
|  |  | 5 | | ≥20 | | 0.961 | 27.5 | | 16.3 | | 0.781 | 23.4 | | 7.8 | | 0.781 | 23.4 | | 7.8 | | 0.750 | 24.3 | 15.9 |
|  |  | 4 | | ≥16 | | 0.996 | 27.1 | | 20.2 | | 0.973 | 22.2 | | 12.4 | | 0.973 | 22.2 | | 12.4 | | 0.940 | 22.7 | 24.4 |
|  |  | 3 | | ≥12 | | 1.000 | 27.0 | | 21.0 | | 1.000 | 22.0 | | 14.0 | | 1.000 | 22.0 | | 14.0 | | 1.000 | 22.0 | 29.9 |
|  |  | 2 | | ≥8 | | 1.000 | 27.0 | | 21.0 | | 1.000 | 22.0 | | 14.0 | | 1.000 | 22.0 | | 14.0 | | 1.000 | 22.0 | 29.9 |
|  |  | 1 | | ≥4 | | 1.000 | 27.0 | | 21.0 | | 1.000 | 22.0 | | 14.0 | | 1.000 | 22.0 | | 14.0 | | 1.000 | 22.0 | 29.9 |
| **2** |  | 0 | |  | | 1.000 | 27.0 | | 21.0 | | 1.000 | 22.0 | | 14.0 | | 1.000 | 22.0 | | 14.0 | | 1.000 | 22.0 | 29.9 |
|  |  | 1 | | <32 | | 0.684 | 24.7 | | 13.8 | | 0.996 | 22.0 | | 13.7 | | 0.996 | 22.0 | | 13.7 | | 0.940 | 21.3 | 24.4 |
|  |  | 2 | | <28 | | 0.367 | 21.8 | | 8.1 | | 0.906 | 21.3 | | 10.3 | | 0.906 | 21.3 | | 10.3 | | 0.750 | 19.7 | 15.9 |
|  | NS | 3 | | <24 | | 0.156 | 18.9 | | 4.0 | | 0.602 | 19.5 | | 5.5 | | 0.602 | 19.5 | | 5.5 | | 0.500 | 17.5 | 8.9 |
|  |  | 4 | | <20 | | 0.039 | 15.6 | | 1.4 | | 0.039 | 16.7 | | 2.3 | | 0.039 | 16.7 | | 2.3 | | 0.250 | 15.0 | 4.0 |
|  |  | 5 | | <16 | | 0.004 | 12.0 | | 0.0 | | 0.027 | 13.7 | | 0.5 | | 0.027 | 13.7 | | 0.5 | | 0.060 | 12.0 | 0.0 |
|  |  | 6 | | <12 | | 0.000 | 0.0 | | 0.0 | | 0.000 | 0.0 | | 0.0 | | 0.000 | 0.0 | | 0.0 | | 0.000 | 0.0 | 0.0 |
|  |  | 7 | | <8 | | 0.000 | 0.0 | | 0.0 | | 0.000 | 0.0 | | 0.0 | | 0.000 | 0.0 | | 0.0 | | 0.000 | 0.0 | 0.0 |
|  |  | 8 | | <4 | | 0.000 | 0.0 | | 0.0 | | 0.000 | 0.0 | | 0.0 | | 0.000 | 0.0 | | 0.0 | | 0.000 | 0.0 | 0.0 |
|  |  |  | |  | |  |  | |  | |  |  | |  | |  |  | |  | |  |  |  |
|  |  | 8 | | ≥32 | | 0.000 | 0.0 | | 0.0 | | 0.000 | 0.0 | | 0.0 | | 0.000 | 0.0 | | 0.0 | | 0.130 | 24.0 | 0.0 |
|  |  | 7 | | ≥28 | | 0.000 | 0.0 | | 0.0 | | 0.000 | 0.0 | | 0.0 | | 0.000 | 0.0 | | 0.0 | | 0.130 | 24.0 | 0.0 |
|  | PS | 6 | | ≥24 | | 0.422 | 24.0 | | 0.0 | | 0.422 | 24.0 | | 0.0 | | 0.016 | 24.0 | | 0.0 | | 0.130 | 24.0 | 0.0 |
|  |  | 5 | | ≥20 | | 0.422 | 24.0 | | 0.0 | | 0.422 | 24.0 | | 0.0 | | 0.047 | 21.3 | | 3.6 | | 0.130 | 24.0 | 0.0 |
|  |  | 4 | | ≥16 | | 0.563 | 22.0 | | 12.0 | | 0.563 | 22.0 | | 12.0 | | 0.094 | 18.7 | | 8.9 | | 0.250 | 20.0 | 32.0 |
|  |  | 3 | | ≥12 | | 0.563 | 22.0 | | 12.0 | | 0.563 | 22.0 | | 12.0 | | 0.188 | 15.3 | | 15.6 | | 0.250 | 20.0 | 32.0 |
|  |  | 2 | | ≥8 | | 0.703 | 19.2 | | 41.0 | | 0.703 | 19.2 | | 41.0 | | 0.391 | 11.8 | | 19.2 | | 0.380 | 16.0 | 64.0 |
|  |  | 1 | | ≥4 | | 0.703 | 19.2 | | 41.0 | | 0.703 | 19.2 | | 41.0 | | 0.641 | 9.2 | | 23.2 | | 0.380 | 16.0 | 64.0 |
| **3** |  | 0 | |  | | 1.000 | 20.3 | | 84.2 | | 1.000 | 16.5 | | 56.3 | | 1.000 | 11.0 | | 35.8 | | 1.000 | 11.0 | 76.2 |
|  |  | 1 | | <32 | | 0.822 | 17.7 | | 66.1 | | 0.998 | 16.5 | | 55.8 | | 1.000 | 11.0 | | 35.7 | | 0.980 | 10.7 | 70.2 |
|  |  | 2 | | <28 | | 0.644 | 14.9 | | 46.9 | | 0.947 | 15.8 | | 50.6 | | 0.994 | 10.9 | | 34.1 | | 0.940 | 9.8 | 57.7 |
|  | NS | 3 | | <24 | | 0.525 | 12.8 | | 34.4 | | 0.776 | 13.8 | | 39.8 | | 0.974 | 10.6 | | 30.7 | | 0.880 | 8.8 | 46.2 |
|  |  | 4 | | <20 | | 0.459 | 11.8 | | 30.8 | | 0.561 | 11.1 | | 26.9 | | 0.910 | 9.9 | | 24.8 | | 0.810 | 7.9 | 39.2 |
|  |  | 5 | | <16 | | 0.229 | 7.5 | | 25.6 | | 0.429 | 9.2 | | 20.4 | | 0.771 | 8.6 | | 17.5 | | 0.640 | 5.8 | 27.2 |
|  |  | 6 | | <12 | | 0.121 | 3.5 | | 14.2 | | 0.238 | 6.3 | | 16.6 | | 0.535 | 6.5 | | 10.9 | | 0.440 | 2.9 | 12.9 |
|  |  | 7 | | <8 | | 0.074 | 0.6 | | 2.1 | | 0.098 | 1.9 | | 6.9 | | 0.315 | 4.3 | | 5.9 | | 0.310 | 0.8 | 2.7 |
|  |  | 8 | | <4 | | 0.063 | 0.0 | | 0.0 | | 0.063 | 0.0 | | 0.0 | | 0.086 | 0.7 | | 1.5 | | 0.250 | 0.0 | 0.0 |
|  |  |  | |  | |  |  | |  | |  |  | |  | |  |  | |  | |  |  |  |
|  |  | 8 | | ≥32 | | 0.133 | 32.0 | | 0.0 | | 0.002 | 32.0 | | 0.0 | | 0.000 | 32.0 | | 0.0 | | 0.010 | 32.0 | 0.0 |
|  |  | 7 | | ≥28 | | 0.267 | 30.0 | | 4.0 | | 0.040 | 28.7 | | 1.2 | | 0.001 | 28.7 | | 1.2 | | 0.030 | 29.0 | 4.0 |
|  | PS | 6 | | ≥24 | | 0.356 | 28.5 | | 9.8 | | 0.168 | 25.7 | | 3.7 | | 0.006 | 25.7 | | 3.7 | | 0.060 | 26.5 | 8.9 |
|  |  | 5 | | ≥20 | | 0.405 | 27.5 | | 16.3 | | 0.330 | 23.4 | | 7.8 | | 0.022 | 22.3 | | 6.3 | | 0.090 | 24.3 | 15.9 |
|  |  | 4 | | ≥16 | | 0.578 | 24.0 | | 39.0 | | 0.428 | 22.0 | | 13.4 | | 0.057 | 19.3 | | 9.2 | | 0.180 | 20.3 | 26.1 |
|  |  | 3 | | ≥12 | | 0.659 | 22.6 | | 49.8 | | 0.571 | 19.7 | | 25.9 | | 0.129 | 15.8 | | 14.1 | | 0.280 | 17.3 | 32.9 |
|  |  | 2 | | ≥8 | | 0.694 | 21.8 | | 57.4 | | 0.677 | 18.1 | | 36.1 | | 0.286 | 12.3 | | 17.3 | | 0.340 | 15.6 | 40.1 |
|  |  | 1 | | ≥4 | | 0.703 | 21.6 | | 60.6 | | 0.703 | 17.6 | | 40.6 | | 0.571 | 9.0 | | 20.0 | | 0.380 | 14.7 | 47.2 |
| **4** |  | 0 | |  | | 1.000 | 15.2 | | 140.0 | | 1.000 | 12.4 | | 93.2 | | 1.000 | 5.5 | | 28.5 | | 1.000 | 5.5 | 68.3 |
|  |  | 1 | | <32 | | 0.867 | 12.6 | | 111.4 | | 0.998 | 12.3 | | 92.8 | | 0.999 | 5.5 | | 28.5 | | 0.990 | 5.3 | 63.2 |
|  |  | 2 | | <28 | | 0.733 | 9.8 | | 80.6 | | 0.960 | 11.7 | | 85.6 | | 0.999 | 5.5 | | 27.8 | | 0.970 | 4.7 | 51.9 |
|  | NS | 3 | | <24 | | 0.644 | 7.8 | | 59.9 | | 0.832 | 9.7 | | 68.0 | | 0.994 | 5.4 | | 26.0 | | 0.940 | 4.1 | 40.7 |
|  |  | 4 | | <20 | | 0.595 | 6.8 | | 51.6 | | 0.670 | 6.9 | | 45.5 | | 0.978 | 5.1 | | 22.4 | | 0.910 | 3.6 | 33.1 |
|  |  | 5 | | <16 | | 0.422 | 3.0 | | 24.0 | | 0.572 | 5.2 | | 32.4 | | 0.943 | 4.7 | | 17.5 | | 0.820 | 2.2 | 18.4 |
|  |  | 6 | | <12 | | 0.341 | 0.9 | | 6.2 | | 0.429 | 2.6 | | 16.6 | | 0.871 | 4.0 | | 12.5 | | 0.720 | 0.9 | 5.6 |
|  |  | 7 | | <8 | | 0.306 | 0.1 | | 0.4 | | 0.323 | 0.4 | | 2.2 | | 0.714 | 2.8 | | 7.2 | | 0.660 | 0.2 | 0.7 |
|  |  | 8 | | <4 | | 0.297 | 0.0 | | 0.0 | | 0.297 | 0.0 | | 0.0 | | 0.429 | 0.8 | | 1.7 | | 0.630 | 0.0 | 0.0 |
|  |  |  | |  | |  |  | |  | |  |  | |  | |  |  | |  | |  |  |  |
|  |  | 8 | | ≥32 | | 0.237 | 32.0 | | 0.0 | | 0.237 | 32.0 | | 0.0 | | 0.001 | 32.0 | | 0.0 | | 0.030 | 32.0 | 0.0 |
|  |  | 7 | | ≥28 | | 0.237 | 32.0 | | 0.0 | | 0.237 | 32.0 | | 0.0 | | 0.001 | 32.0 | | 0.0 | | 0.340 | 17.5 | 21.0 |
|  | PS | 6 | | ≥24 | | 0.237 | 32.0 | | 0.0 | | 0.237 | 32.0 | | 0.0 | | 0.011 | 24.7 | | 5.3 | | 0.340 | 17.5 | 21.0 |
|  |  | 5 | | ≥20 | | 0.237 | 32.0 | | 0.0 | | 0.237 | 32.0 | | 0.0 | | 0.026 | 21.9 | | 7.6 | | 0.340 | 17.5 | 21.0 |
|  |  | 4 | | ≥16 | | 0.747 | 21.1 | | 55.5 | | 0.747 | 21.1 | | 55.5 | | 0.101 | 17.7 | | 8.6 | | 0.340 | 17.5 | 21.0 |
|  |  | 3 | | ≥12 | | 0.747 | 21.1 | | 55.5 | | 0.747 | 21.1 | | 55.5 | | 0.171 | 15.4 | | 12.9 | | 0.340 | 17.5 | 21.0 |
|  |  | 2 | | ≥8 | | 0.747 | 21.1 | | 55.5 | | 0.747 | 21.1 | | 55.5 | | 0.435 | 11.0 | | 17.5 | | 0.340 | 17.5 | 21.0 |
|  |  | 1 | | ≥4 | | 0.747 | 21.1 | | 55.5 | | 0.747 | 21.1 | | 55.5 | | 0.692 | 8.5 | | 21.9 | | 0.340 | 17.5 | 21.0 |
| **5** |  | 0 | |  | | 1.000 | 11.8 | | 140.6 | | 1.000 | 11.8 | | 140.6 | | 1.000 | 3.0 | | 15.6 | | 1.000 | 3.0 | 47.0 |
|  |  | 1 | | <32 | | 0.822 | 7.4 | | 63.7 | | 0.822 | 7.4 | | 63.7 | | 1.000 | 3.0 | | 15.4 | | 0.980 | 2.5 | 34.2 |
|  |  | 2 | | <28 | | 0.822 | 7.4 | | 63.7 | | 0.822 | 7.4 | | 63.7 | | 1.000 | 3.0 | | 15.4 | | 0.980 | 2.5 | 34.2 |
|  | NS | 3 | | <24 | | 0.822 | 7.4 | | 63.7 | | 0.822 | 7.4 | | 63.7 | | 0.997 | 2.9 | | 14.3 | | 0.980 | 2.5 | 34.2 |
|  |  | 4 | | <20 | | 0.822 | 7.4 | | 63.7 | | 0.822 | 7.4 | | 63.7 | | 0.993 | 2.9 | | 13.2 | | 0.980 | 2.5 | 34.2 |
|  |  | 5 | | <16 | | 0.440 | 0.0 | | 0.0 | | 0.440 | 0.0 | | 0.0 | | 0.974 | 2.6 | | 9.9 | | 0.830 | 0.0 | 0.0 |
|  |  | 6 | | <12 | | 0.440 | 0.0 | | 0.0 | | 0.440 | 0.0 | | 0.0 | | 0.952 | 2.4 | | 8.0 | | 0.830 | 0.0 | 0.0 |
|  |  | 7 | | <8 | | 0.440 | 0.0 | | 0.0 | | 0.440 | 0.0 | | 0.0 | | 0.841 | 1.6 | | 3.8 | | 0.830 | 0.0 | 0.0 |
|  |  | 8 | | <4 | | 0.440 | 0.0 | | 0.0 | | 0.440 | 0.0 | | 0.0 | | 0.610 | 0.5 | | 0.8 | | 0.830 | 0.0 | 0.0 |
|  |  |  | |  | |  |  | |  | |  |  | |  | |  |  | |  | |  |  |  |
|  |  | 8 | | ≥32 | | 0.237 | 32.0 | | 0.0 | | 0.001 | 32.0 | | 0.0 | | 0.001 | 32.0 | | 0.0 | | 0.030 | 32.0 | 0.0 |
|  |  | 7 | | ≥28 | | 0.422 | 30.3 | | 3.9 | | 0.021 | 28.7 | | 1.1 | | 0.021 | 28.7 | | 1.1 | | 0.130 | 29.0 | 3.0 |
|  | PS | 6 | | ≥24 | | 0.747 | 27.5 | | 11.8 | | 0.212 | 25.0 | | 2.3 | | 0.212 | 25.0 | | 2.3 | | 0.340 | 25.8 | 6.9 |
|  |  | 5 | | ≥20 | | 1.000 | 25.6 | | 19.6 | | 1.000 | 22.0 | | 3.6 | | 1.000 | 22.0 | | 3.6 | | 0.340 | 25.8 | 6.9 |
|  |  | 4 | | ≥16 | | 1.000 | 25.6 | | 19.6 | | 1.000 | 22.0 | | 3.6 | | 1.000 | 22.0 | | 3.6 | | 0.340 | 25.8 | 6.9 |
|  |  | 3 | | ≥12 | | 1.000 | 25.6 | | 19.6 | | 1.000 | 22.0 | | 3.6 | | 1.000 | 22.0 | | 3.6 | | 0.340 | 25.8 | 6.9 |
|  |  | 2 | | ≥8 | | 1.000 | 25.6 | | 19.6 | | 1.000 | 22.0 | | 3.6 | | 1.000 | 22.0 | | 3.6 | | 0.340 | 25.8 | 6.9 |
|  |  | 1 | | ≥4 | | 1.000 | 25.6 | | 19.6 | | 1.000 | 22.0 | | 3.6 | | 1.000 | 22.0 | | 3.6 | | 0.340 | 25.8 | 6.9 |
| **6** |  | 0 | |  | | 1.000 | 19.2 | | 77.0 | | 1.000 | 16.5 | | 49.6 | | 1.000 | 11.0 | | 32.6 | | 1.000 | 11.0 | 68.0 |
|  |  | 1 | | <32 | | 0.870 | 17.2 | | 59.8 | | 0.999 | 16.5 | | 49.5 | | 0.999 | 11.0 | | 32.6 | | 0.990 | 10.8 | 65.0 |
|  |  | 2 | | <28 | | 0.760 | 15.8 | | 50.1 | | 0.988 | 16.4 | | 48.3 | | 0.999 | 11.0 | | 32.2 | | 0.970 | 10.4 | 59.0 |
|  | NS | 3 | | <24 | | 0.580 | 13.1 | | 37.9 | | 0.881 | 15.4 | | 45.0 | | 0.987 | 10.8 | | 30.3 | | 0.910 | 9.6 | 51.0 |
|  |  | 4 | | <20 | | 0.440 | 11.0 | | 30.2 | | 0.438 | 9.4 | | 19.8 | | 0.922 | 10.1 | | 24.5 | | 0.750 | 7.3 | 33.0 |
|  |  | 5 | | <16 | | 0.250 | 7.3 | | 20.5 | | 0.423 | 9.2 | | 18.9 | | 0.757 | 8.6 | | 16.9 | | 0.660 | 6.1 | 25.0 |
|  |  | 6 | | <12 | | 0.170 | 5.1 | | 14.8 | | 0.229 | 6.2 | | 15.0 | | 0.546 | 6.8 | | 11.6 | | 0.470 | 3.7 | 16.0 |
|  |  | 7 | | <8 | | 0.060 | 0.0 | | 0.0 | | 0.063 | 0.0 | | 0.0 | | 0.303 | 4.3 | | 5.8 | | 0.250 | 0.0 | 0.0 |
|  |  | 8 | | <4 | | 0.060 | 0.0 | | 0.0 | | 0.063 | 0.0 | | 0.0 | | 0.063 | 0.0 | | 0.0 | | 0.250 | 0.0 | 0.0 |
|  |  |  | |  | |  |  | |  | |  |  | |  | |  |  | |  | |  |  |  |
|  |  | 8 | | ≥32 | | 0.100 | 32.0 | | 0.0 | | 0.000 | 32.0 | | 0.0 | | 0.000 | 32.0 | | 0.0 | | 0.004 | 32.0 | 0.0 |
|  |  | 7 | | ≥28 | | 0.200 | 30.0 | | 4.0 | | 0.000 | 30.3 | | 0.5 | | 0.000 | 28.5 | | 1.0 | | 0.016 | 29.0 | 3.0 |
|  | PS | 6 | | ≥24 | | 0.404 | 27.0 | | 11.0 | | 0.017 | 24.8 | | 1.9 | | 0.006 | 24.8 | | 1.9 | | 0.060 | 25.3 | 5.7 |
|  |  | 5 | | ≥20 | | 0.578 | 24.9 | | 17.9 | | 0.117 | 21.3 | | 3.2 | | 0.046 | 21.2 | | 2.9 | | 0.160 | 22.0 | 8.8 |
|  |  | 4 | | ≥16 | | 0.822 | 22.3 | | 29.0 | | 0.377 | 18.3 | | 5.9 | | 0.235 | 17.8 | | 4.2 | | 0.400 | 18.3 | 12.0 |
|  |  | 3 | | ≥12 | | 0.921 | 21.1 | | 36.0 | | 0.713 | 15.9 | | 10.3 | | 0.602 | 15.0 | | 7.3 | | 0.640 | 16.0 | 16.9 |
|  |  | 2 | | ≥8 | | 0.983 | 20.3 | | 43.9 | | 0.938 | 14.3 | | 15.9 | | 0.908 | 13.2 | | 12.0 | | 0.850 | 14.0 | 24.5 |
|  |  | 1 | | ≥4 | | 1.000 | 20.0 | | 47.7 | | 1.000 | 13.8 | | 19.5 | | 1.000 | 12.5 | | 15.5 | | 1.000 | 12.5 | 33.8 |
| **7** |  | 0 | |  | | 1.000 | 20.0 | | 47.7 | | 1.000 | 13.8 | | 19.5 | | 1.000 | 12.5 | | 15.5 | | 1.000 | 12.5 | 33.8 |
|  |  | 1 | | <32 | | 0.900 | 18.7 | | 35.3 | | 1.000 | 13.8 | | 19.5 | | 1.000 | 12.5 | | 15.5 | | 0.996 | 12.4 | 32.4 |
|  |  | 2 | | <28 | | 0.800 | 17.5 | | 27.5 | | 0.999 | 13.7 | | 19.2 | | 1.000 | 12.5 | | 15.4 | | 0.984 | 12.2 | 29.8 |
|  | NS | 3 | | <24 | | 0.596 | 15.3 | | 17.7 | | 0.983 | 13.6 | | 17.6 | | 0.994 | 12.4 | | 14.7 | | 0.941 | 11.7 | 24.6 |
|  |  | 4 | | <20 | | 0.422 | 13.4 | | 12.3 | | 0.883 | 12.7 | | 13.0 | | 0.954 | 12.1 | | 12.3 | | 0.844 | 10.7 | 18.6 |
|  |  | 5 | | <16 | | 0.178 | 9.8 | | 7.1 | | 0.623 | 11.0 | | 7.6 | | 0.765 | 10.9 | | 7.7 | | 0.598 | 8.6 | 10.1 |
|  |  | 6 | | <12 | | 0.079 | 7.1 | | 2.7 | | 0.287 | 8.5 | | 3.5 | | 0.398 | 8.7 | | 3.6 | | 0.359 | 6.3 | 3.9 |
|  |  | 7 | | <8 | | 0.017 | 4.0 | | 0.0 | | 0.006 | 5.5 | | 0.9 | | 0.092 | 5.8 | | 1.1 | | 0.152 | 4.0 | 0.0 |
|  |  | 8 | | <4 | | 0.000 | 0.0 | | 0.0 | | 0.000 | 0.0 | | 0.0 | | 0.000 | 0.0 | | 0.0 | | 0.000 | 0.0 | 0.0 |

^1^ TT = the threshold trait value in the selection; PS and NS = positive and negative selection for increased and decreased trait values, respectively.

^2^ S.I. = selection intensity.
