# Supplementary material for: Dissecting Genetic Networks Underlying Complex Phenotypes: The Theoretical Framework
Source: PLoS One. 2011 Jan 20;6(1):e14541. doi: 10.1371/journal.pone.0014541 (PMC3024316; doi:10.1371/journal.pone.0014541)
Supplement: Table S10 — Nonrandom associations, measured by the normalized gametic linkage disequilibrium statistics (LD'), between loci segregating in a signaling pathway of model (2) resulting from positive and negative selection for increased trait values under the seven scenarios of an RI (or DH) populations defined in Table 1. (0.26 MB DOC) [file pone.0014541.s010.doc]

**Table S10. Non-random associations, measured by the normalized gametic linkage disequilibrium statistics (LD’), between loci segregating in a signaling pathway of model (2) resulting from positive and negative selection for increased trait values under the 7 scenarios of a RI (or DH) populations defined in Table 1**

| Scenario | M_i_ | M_j_ | Selection steps (negative) ^1^ | | | | | | | |  | Selection steps (positive) ^1^ | | | | | | | |
| --- | --- | --- | --- | --- | --- | --- | --- | --- | --- | --- | --- | --- | --- | --- | --- | --- | --- | --- | --- |
|  |  |  | 8 | 7 | 6 | 5 | 4 | 3 | 2 | 1 | 0 | 1 | 2 | 3 | 4 | 5 | 6 | 7 | 8 |
| **1** | T_1_ | T_2_ |  |  | NC | NC |  |  | -1.00 |  | 0.00 |  |  | -1.00 | NC |  |  | NC |  |
| **2** | B_11_ | B_13_ |  |  |  | NC | -1.00 | -0.11 | -0.04 | -0.08 | 0.00 |  |  |  | -0.08 | -0.04 | -0.11 | -1.00 | NC |
|  | B_11_ | B_21_ |  |  |  | NC | NC | -1.00 | -0.40 | -0.08 | 0.00 |  |  |  | -0.08 | -0.40 | -1.00 | NC | NC |
|  | B_11_ | B_23_ |  |  |  | NC | -1.00 | -0.11 | -0.04 | -0.08 | 0.00 |  |  |  | -0.08 | -0.04 | -0.11 | -1.00 | NC |
|  | B_13_ | B_21_ |  |  |  | NC | NC | -1.00 | -0.40 | -0.08 | 0.00 |  |  |  | -0.08 | -0.40 | -1.00 | NC | NC |
|  | B_13_ | B_23_ |  |  |  | NC | -1.00 | -0.11 | -0.04 | -0.08 | 0.00 |  |  |  | -0.08 | -0.04 | -0.11 | -1.00 | NC |
|  | B_21_ | B_23_ |  |  |  | NC | NC | -1.00 | -0.40 | -0.08 | 0.00 |  |  |  | -0.08 | -0.40 | -1.00 | NC | NC |
| **3** | **T_1_** | **B_11_** | NC | NC | -1.00** | -0.30 | -0.06 | -0.05 | -0.04 | -0.02 | 0.00 | 0.00 | 0.00 | **0.10** | **0.18** | NC | NC | NC | NC |
|  | **T_1_** | **B_13_** | NC | NC | -1.00** | -0.30 | -0.06 | -0.05 | -0.04 | -0.02 | 0.00 | 0.00 | 0.00 | **0.10** | **0.18** | NC | NC | NC | NC |
|  | T_1_ | T_2_ | NC | NC | -1.00 | -0.76** | -0.48** | -0.22 | -0.08 | -0.02 | 0.00 | -1.00** | -1.00** | -1.00** | -1.00 | NC | NC | -1.00 | NC |
|  | T_1_ | B_21_ | NC | NC | 0.22 | 0.04 | -0.13 | -0.16 | -0.08 | -0.02 | 0.00 | 0.00 | -0.27 | -1.00** | -1.00* | NC | NC | NC | NC |
|  | T_1_ | B_23_ | NC | NC | 0.00 | 0.04 | -0.06 | -0.05 | -0.04 | -0.02 | 0.00 | 0.00 | -0.27 | 0.00 | -1.00* | NC | NC | NC | NC |
|  | B_11_ | B_13_ | 0.00 | 0.00 | 0.13 | -0.11 | 0.00 | 0.00 | 0.00 | -0.02 | 0.00 | 0.00 | 0.00 | -0.16 | 0.09 | -0.04 | -0.11 | -1.00 | NC |
|  | B_11_ | T_2_ | NC | 0.00 | 0.13 | 0.04 | -0.06 | -0.05 | -0.04 | -0.02 | 0.00 | 0.00 | 0.00 | -0.25 | -1.00* | NC | NC | NC | NC |
|  | B_11_ | B_21_ | 0.00 | 0.00 | -0.07 | 0.01 | 0.04 | 0.00 | -0.04 | -0.02 | 0.00 | 0.00 | 0.00 | -0.04 | -0.15 | -0.40 | -1.00 | NC | NC |
|  | B_11_ | B_23_ | 0.00 | 0.00 | 0.00 | 0.01 | 0.00 | 0.00 | 0.00 | -0.02 | 0.00 | 0.00 | 0.00 | 0.00 | -0.15 | -0.04 | -0.11 | -1.00 | NC |
|  | B_13_ | T_2_ | NC | 0.00 | 0.13 | 0.04 | -0.06 | -0.05 | -0.04 | -0.02 | 0.00 | 0.00 | 0.00 | -0.25 | -1.00* | NC | NC | NC | NC |
|  | B_13_ | B_21_ | 0.00 | 0.00 | -0.07 | 0.01 | 0.04 | 0.00 | -0.04 | -0.02 | 0.00 | 0.00 | 0.00 | -0.04 | -0.15 | -0.40 | -1.00 | NC | NC |
|  | B_13_ | B_23_ | 0.00 | 0.00 | 0.00 | 0.01 | 0.00 | 0.00 | 0.00 | -0.02 | 0.00 | 0.00 | 0.00 | 0.00 | -0.15 | -0.04 | -0.11 | NC | NC |
|  | **T_2_** | **B_21_** | NC | -1.00** | -1.00** | -0.30 | -0.13 | -0.16 | -0.08 | -0.02 | 0.00 | 0.00 | **0.08** | **0.18** | **0.18** | NC | NC | NC | NC |
|  | **T_2_** | **B_23_** | NC | -1.00** | 0.00 | -0.30 | -0.06 | -0.05 | -0.04 | -0.02 | 0.00 | 0.00 | **0.08** | **0.00** | **0.18** | NC | NC | NC | NC |
|  | B_21_ | B_23_ | 0.00 | 0.17 | 0.00 | -0.11 | 0.04 | 0.00 | -0.04 | -0.02 | 0.00 | 0.00 | -0.12 | 0.00 | 0.09 | -0.40 | -1.00 | NC | NC |
| **4** | S | T_1_ | -1.00** | -1.00** | -0.63** | -0.26** | -0.14 | -0.08 | -0.04 | -0.01 | 0.00 | NC | NC | NC | NC | NC | NC | NC | NC |
|  | S | B_11_ | 0.00 | 0.00 | -0.10 | -0.08 | -0.02 | -0.02 | -0.02 | -0.01 | 0.00 | NC | NC | NC | NC | NC | NC | NC | NC |
|  | S | B_13_ | 0.00 | 0.00 | -0.10 | -0.08 | -0.02 | -0.02 | -0.02 | -0.01 | 0.00 | NC | NC | NC | NC | NC | NC | NC | NC |
|  | S | T_2_ | -1.00* | -0.53** | -0.34** | -0.26** | -0.14 | -0.08 | -0.04 | -0.01 | 0.00 | NC | NC | NC | NC | NC | NC | NC | NC |
|  | S | B_21_ | 0.00 | -0.16 | -0.22 | -0.08 | -0.04 | -0.06 | -0.04 | -0.01 | 0.00 | NC | NC | NC | NC | NC | NC | NC | NC |
|  | S | B_23_ | 0.00 | -0.16 | 0.00 | -0.08 | -0.02 | -0.02 | -0.02 | -0.01 | 0.00 | NC | NC | NC | NC | NC | NC | NC | NC |
|  | T_1_ | B_11_ | 0.00 | 0.00 | -0.07 | -0.07 | -0.02 | -0.02 | -0.02 | -0.01 | 0.00 | 0.00 | 0.00 | **0.10** | **0.18** | NC | NC | NC | NC |
|  | T_1_ | B_13_ | 0.00 | 0.00 | -0.07 | -0.07 | -0.02 | -0.02 | -0.02 | -0.01 | 0.00 | 0.00 | 0.00 | **0.10** | **0.18** | NC | NC | NC | NC |
|  | T_1_ | T_2_ | 0.17 | 0.13 | 0.02 | -0.12 | -0.14 | -0.08 | -0.04 | -0.01 | 0.00 | -1.00** | -1.00** | -1.00** | -1.00 | NC | NC | NC | NC |
|  | T_1_ | B_21_ | 0.00 | 0.05 | 0.08 | 0.02 | -0.04 | -0.06 | -0.04 | -0.01 | 0.00 | 0.00 | -0.27 | -1.00** | -1.00* | NC | NC | NC | NC |
|  | T_1_ | B_23_ | 0.00 | 0.05 | 0.00 | 0.02 | -0.02 | -0.02 | -0.02 | -0.01 | 0.00 | 0.00 | -0.27 | 0.00 | -1.00* | NC | NC | NC | NC |
|  | B_11_ | B_13_ | 0.00 | 0.00 | 0.04 | -0.03 | 0.00 | 0.00 | 0.00 | -0.01 | 0.00 | 0.00 | 0.00 | -0.16 | 0.09 | -0.04 | -0.11 | -1.00 | NC |
|  | B_11_ | T_2_ | 0.00 | 0.00 | 0.04 | 0.02 | -0.02 | -0.02 | -0.02 | -0.01 | 0.00 | 0.00 | 0.00 | -0.25 | -1.00* | NC | NC | NC | NC |
|  | B_11_ | B_21_ | 0.00 | 0.00 | 0.00 | 0.01 | 0.02 | 0.00 | -0.02 | -0.01 | 0.00 | 0.00 | 0.00 | -0.04 | -0.15 | -0.40 | -1.00 | NC | NC |
|  | B_11_ | B_23_ | 0.00 | 0.00 | 0.00 | 0.01 | 0.00 | 0.00 | 0.00 | -0.01 | 0.00 | 0.00 | 0.00 | 0.00 | -0.15 | -0.04 | -0.11 | -1.00 | NC |
|  | B_13_ | T_2_ | 0.00 | 0.00 | 0.04 | 0.02 | -0.02 | -0.02 | -0.02 | -0.01 | 0.00 | 0.00 | 0.00 | -0.25 | -1.00* | NC | NC | NC | NC |
|  | B_13_ | B_21_ | 0.00 | 0.00 | 0.00 | 0.01 | 0.02 | 0.00 | -0.02 | -0.01 | 0.00 | 0.00 | 0.00 | -0.04 | -0.15 | -0.40 | -1.00 | NC | NC |
|  | B_13_ | B_23_ | 0.00 | 0.00 | 0.00 | 0.01 | 0.00 | 0.00 | 0.00 | -0.01 | 0.00 | 0.00 | 0.00 | 0.00 | -0.15 | -0.04 | -0.11 | -1.00 | NC |
|  | T_2_ | B_21_ | 0.00 | -0.07 | -0.12 | -0.07 | -0.04 | -0.06 | -0.04 | -0.01 | 0.00 | 0.00 | **0.08** | **0.18** | **0.18** | NC | NC | NC | NC |
|  | T_2_ | B_23_ | 0.00 | -0.07 | 0.00 | -0.07 | -0.02 | -0.02 | -0.02 | -0.01 | 0.00 | 0.00 | **0.08** | **0.00** | **0.18** | NC | NC | NC | NC |
|  | B_21_ | B_23_ | 0.00 | 0.05 | 0.00 | -0.03 | 0.02 | 0.00 | -0.02 | -0.01 | 0.00 | 0.00 | -0.12 | 0.00 | 0.09 | -0.40 | -1.00 | NC | NC |
| **5** | S | T_11_ |  |  |  | -0.23 |  |  |  | -0.02 | 0.00 |  |  |  | NC |  |  |  | NC |
|  | S | T_12_ |  |  |  | -0.23 |  |  |  | -0.02 | 0.00 |  |  |  | NC |  |  |  | NC |
|  | S | T_21_ |  |  |  | -0.09 |  |  |  | -0.02 | 0.00 |  |  |  | NC |  |  |  | NC |
|  | S | T_22_ |  |  |  | -0.09 |  |  |  | -0.02 | 0.00 |  |  |  | NC |  |  |  | NC |
|  | S | T_23_ |  |  |  | -0.09 |  |  |  | -0.02 | 0.00 |  |  |  | NC |  |  |  | NC |
|  | T_11_ | T_12_ |  |  |  | -0.20 |  |  |  | -0.02 | 0.00 |  |  |  | **0.39** |  |  |  | NC |
|  | T_11_ | T_21_ |  |  |  | 0.01 |  |  |  | -0.02 | 0.00 |  |  |  | -1.00 |  |  |  | NC |
|  | T_11_ | T_22_ |  |  |  | 0.01 |  |  |  | -0.02 | 0.00 |  |  |  | -1.00 |  |  |  | NC |
|  | T_11_ | T_23_ |  |  |  | 0.01 |  |  |  | -0.02 | 0.00 |  |  |  | -1.00 |  |  |  | NC |
|  | T_12_ | T_21_ |  |  |  | 0.01 |  |  |  | -0.02 | 0.00 |  |  |  | -1.00 |  |  |  | NC |
|  | T_12_ | T_22_ |  |  |  | 0.01 |  |  |  | -0.02 | 0.00 |  |  |  | -1.00 |  |  |  | NC |
|  | T_12_ | T_23_ |  |  |  | 0.01 |  |  |  | -0.02 | 0.00 |  |  |  | -1.00 |  |  |  | NC |
|  | T_21_ | T_22_ |  |  |  | -0.07 |  |  |  | -0.02 | 0.00 |  |  |  | **0.21** |  |  |  | NC |
|  | T_21_ | T_23_ |  |  |  | -0.07 |  |  |  | -0.02 | 0.00 |  |  |  | **0.21** |  |  |  | NC |
|  | T_22_ | T_23_ |  |  |  | -0.07 |  |  |  | -0.02 | 0.00 |  |  |  | **0.21** |  |  |  | NC |
| **6** | T_1_ | B_111_ |  | NC | NC | -0.26* | 0.00 | -0.08 | 0.00 | -0.01 | 0.00 |  | 0.00 | 0.00 | **0.15** | NC | NC | NC | NC |
|  | T_1_ | B_112_ |  | NC | NC | -0.26* | 0.00 | -0.08 | 0.00 | -0.01 | 0.00 |  | 0.00 | 0.00 | **0.15** | NC | NC | NC | NC |
|  | T_1_ | T_2_ |  | NC | NC | -1.00** | -1.00** | -0.13 | -0.04 | -0.01 | 0.00 |  | -1.00** | -1.00** | -1.00 | NC | NC | NC | NC |
|  | T_1_ | B_211_ |  | NC | NC | 0.05 | 0.00 | -0.03 | -0.04 | -0.01 | 0.00 |  | 0.00 | -1.00** | -1.00** | NC | NC | NC | NC |
|  | T_1_ | B_212_ |  | NC | NC | 0.05 | 0.00 | -0.03 | -0.04 | -0.01 | 0.00 |  | 0.00 | -1.00** | -1.00** | NC | NC | NC | NC |
|  | T_1_ | B_213_ |  | NC | NC | 0.05 | 0.00 | -0.03 | -0.04 | -0.01 | 0.00 |  | 0.00 | -1.00** | -1.00** | NC | NC | NC | NC |
|  | B_111_ | B_112_ |  | 0.00 | 0.00 | -0.13 | 0.00 | -0.07 | 0.00 | -0.01 | 0.00 |  | 0.00 | 0.00 | **0.15** | **0.00** | **0.39** | 0.00 | NC |
|  | B_111_ | T_2_ |  | NC | 0.00 | 0.09 | 0.00 | -0.08 | 0.00 | -0.01 | 0.00 |  | 0.00 | 0.00 | -1.00** | NC | NC | NC | NC |
|  | B_111_ | B_211_ |  | 0.00 | 0.00 | -0.01 | 0.00 | 0.01 | 0.00 | -0.01 | 0.00 |  | 0.00 | 0.00 | -0.02 | 0.00 | -1.00 | NC | NC |
|  | B_111_ | B_212_ |  | 0.00 | 0.00 | -0.01 | 0.00 | 0.01 | 0.00 | -0.01 | 0.00 |  | 0.00 | 0.00 | -0.02 | 0.00 | -1.00 | NC | NC |
|  | B_111_ | B_213_ |  | 0.00 | 0.00 | -0.01 | 0.00 | 0.01 | 0.00 | -0.01 | 0.00 |  | 0.00 | 0.00 | -0.02 | 0.00 | -1.00 | NC | NC |
|  | B_112_ | T2 |  | NC | 0.00 | 0.09 | 0.00 | -0.08 | 0.00 | -0.01 | 0.00 |  | 0.00 | 0.00 | -1.00** | NC | NC | NC | NC |
|  | B_112_ | B_211_ |  | 0.00 | 0.00 | -0.01 | 0.00 | 0.01 | 0.00 | -0.01 | 0.00 |  | 0.00 | 0.00 | -0.02 | 0.00 | -1.00 | NC | NC |
|  | B_112_ | B_212_ |  | 0.00 | 0.00 | -0.01 | 0.00 | 0.01 | 0.00 | -0.01 | 0.00 |  | 0.00 | 0.00 | -0.02 | 0.00 | -1.00 | NC | NC |
|  | B_112_ | B_213_ |  | 0.00 | 0.00 | -0.01 | 0.00 | 0.01 | 0.00 | -0.01 | 0.00 |  | 0.00 | 0.00 | -0.02 | 0.00 | -1.00 | NC | NC |
|  | T_2_ | B_211_ |  | NC | -0.08 | -0.10 | 0.00 | -0.03 | -0.04 | -0.01 | 0.00 |  | 0.00 | **0.06** | **0.08** | NC | NC | NC | NC |
|  | T_2_ | B_212_ |  | NC | -0.08 | -0.10 | 0.00 | -0.03 | -0.04 | -0.01 | 0.00 |  | 0.00 | **0.06** | **0.08** | NC | NC | NC | NC |
|  | T_2_ | B_213_ |  | NC | -0.08 | -0.10 | 0.00 | -0.03 | -0.04 | -0.01 | 0.00 |  | 0.00 | **0.06** | **0.08** | NC | NC | NC | NC |
|  | B_211_ | B_212_ |  | 0.00 | -0.08 | -0.06 | 0.00 | -0.03 | -0.04 | -0.01 | 0.00 |  | 0.00 | **0.06** | **0.08** | 0.00 | 0.21 | NC | NC |
|  | B_211_ | B_213_ |  | 0.00 | -0.08 | -0.06 | 0.00 | -0.03 | -0.04 | -0.01 | 0.00 |  | 0.00 | **0.06** | **0.08** | 0.00 | 0.21 | NC | NC |
|  | B_212_ | B_213_ |  | 0.00 | -0.08 | -0.06 | 0.00 | -0.03 | -0.04 | -0.01 | 0.00 |  | 0.00 | **0.06** | **0.08** | 0.00 | 0.21 | NC | NC |
| **7** | B_12_ | B_13_ |  | NC | NC | -1.00** | -0.08 | -0.03 | -0.01 | 0.00 | 0.00 |  | -0.32** | -0.33** | -0.21 | -0.17 | -1.00 | NC | NC |
|  | B_12_ | T_2_ |  | NC | NC | -0.52** | -0.25** | -0.06 | -0.02 | 0.00 | 0.00 |  | -0.19** | -1.00** | -1.00** | NC | NC | NC | NC |
|  | B_12_ | B_211_ |  | NC | NC | 0.02 | -0.01 | -0.02 | -0.02 | 0.00 | 0.00 |  | -0.01 | -0.09 | -0.39* | -1.00* | -1.00 | NC | NC |
|  | B_12_ | B_212_ |  | NC | NC | 0.02 | -0.01 | -0.02 | -0.02 | 0.00 | 0.00 |  | -0.01 | -0.09 | -0.39* | -1.00* | -1.00 | NC | NC |
|  | B_12_ | B_213_ |  | NC | NC | 0.02 | -0.01 | -0.02 | -0.02 | 0.00 | 0.00 |  | -0.01 | -0.09 | -0.39* | -1.00* | -1.00 | NC | NC |
|  | B_12_ | B_22_ |  | NC | NC | -0.09 | -0.08 | -0.03 | -0.01 | 0.00 | 0.00 |  | -0.05 | -0.33** | -0.50* | -0.17 | -1.00 | NC | NC |
|  | B_12_ | B_23_ |  | NC | NC | -0.09 | -0.08 | -0.03 | -0.01 | 0.00 | 0.00 |  | -0.05 | -0.33** | -0.50* | -0.17 | -1.00 | NC | NC |
|  | B_13_ | T_2_ |  | NC | -0.41** | 0.04 | -0.12 | -0.04 | -0.01 | 0.00 | 0.00 |  | -0.19** | 0.08 | -1.00** | NC | NC | NC | NC |
|  | B_13_ | B_211_ |  | NC | 0.02 | -0.01 | 0.00 | -0.01 | -0.01 | 0.00 | 0.00 |  | -0.01 | -0.02 | -0.08 | -0.17 | -1.00 | NC | NC |
|  | B_13_ | B_212_ |  | NC | 0.02 | -0.01 | 0.00 | -0.01 | -0.01 | 0.00 | 0.00 |  | -0.01 | -0.02 | -0.08 | -0.17 | -1.00 | NC | NC |
|  | B_13_ | B_213_ |  | NC | 0.02 | -0.01 | 0.00 | -0.01 | -0.01 | 0.00 | 0.00 |  | -0.01 | -0.02 | -0.08 | -0.17 | -1.00 | NC | NC |
|  | B_13_ | B_22_ |  | NC | -0.03 | -0.04 | -0.01 | -0.03 | 0.00 | 0.00 | 0.00 |  | -0.05 | -0.01 | -0.16 | -0.44 | 0.17 | -1.00 | NC |
|  | B_13_ | B_23_ |  | NC | -0.03 | -0.04 | -0.01 | -0.03 | 0.00 | 0.00 | 0.00 |  | -0.05 | -0.01 | -0.16 | -0.44 | 0.17 | -1.00 | NC |
|  | T_2_ | B_211_ |  | -0.12 | -0.10 | -0.08 | -0.06 | -0.03 | -0.02 | 0.00 | 0.00 |  | 0.00 | **0.02** | **0.06** | NC | NC | NC | NC |
|  | T_2_ | B_212_ |  | -0.12 | -0.10 | -0.08 | -0.06 | -0.03 | -0.02 | 0.00 | 0.00 |  | 0.00 | **0.02** | **0.06** | NC | NC | NC | NC |
|  | T_2_ | B_213_ |  | -0.12 | -0.10 | -0.08 | -0.06 | -0.03 | -0.02 | 0.00 | 0.00 |  | 0.00 | **0.02** | **0.06** | NC | NC | NC | NC |
|  | T_2_ | B_22_ |  | -1.00** | -0.41** | -0.18* | -0.12 | -0.04 | -0.01 | 0.00 | 0.00 |  | **0.03** | **0.08** | **0.13** | NC | NC | NC | NC |
|  | T_2_ | B_23_ |  | -1.00** | -0.41** | -0.18* | -0.12 | -0.04 | -0.01 | 0.00 | 0.00 |  | **0.03** | **0.08** | **0.13** | NC | NC | NC | NC |
|  | B_211_ | B_212_ |  | -0.03 | -0.05 | -0.05 | -0.04 | -0.03 | -0.02 | 0.00 | 0.00 |  | 0.00 | **0.02** | **0.06** | 0.17 | 0.32 | NC | NC |
|  | B_211_ | B_213_ |  | -0.03 | -0.05 | -0.05 | -0.04 | -0.03 | -0.02 | 0.00 | 0.00 |  | 0.00 | **0.02** | **0.06** | 0.17 | 0.32 | NC | NC |
|  | B_211_ | B_22_ |  | 0.025 | 0.02 | 0.00 | 0.00 | -0.01 | -0.01 | 0.00 | 0.00 |  | 0.00 | -0.02 | -0.02 | -0.17 | -1.00 | NC | NC |
|  | B_211_ | B_23_ |  | 0.025 | 0.02 | 0.00 | 0.00 | -0.01 | -0.01 | 0.00 | 0.00 |  | 0.00 | -0.02 | -0.02 | -0.17 | -1.00 | NC | NC |
|  | B_212_ | B_213_ |  | -0.03 | -0.05 | -0.05 | -0.04 | -0.03 | -0.02 | 0.00 | 0.00 |  | 0.00 | **0.02** | **0.06** | 0.17 | 0.32 | NC | NC |
|  | B_212_ | B_22_ |  | 0.025 | 0.02 | 0.00 | 0.00 | -0.01 | -0.01 | 0.00 | 0.00 |  | 0.00 | -0.02 | -0.02 | -0.17 | -1.00 | NC | NC |
|  | B_212_ | B_23_ |  | 0.025 | 0.02 | 0.00 | 0.00 | -0.01 | -0.01 | 0.00 | 0.00 |  | 0.00 | -0.02 | -0.02 | -0.17 | -1.00 | NC | NC |
|  | B_213_ | B_22_ |  | 0.025 | 0.02 | 0.00 | 0.00 | -0.01 | -0.01 | 0.00 | 0.00 |  | 0.00 | -0.02 | -0.02 | -0.17 | -1.00 | NC | NC |
|  | B_213_ | B_23_ |  | 0.025 | 0.02 | 0.00 | 0.00 | -0.01 | -0.01 | 0.00 | 0.00 |  | 0.00 | -0.02 | -0.02 | -0.17 | -1.00 | NC | NC |
|  | B_22_ | B_23_ |  | 0.152 | -0.03 | 0.00 | -0.01 | -0.03 | 0.00 | 0.00 | 0.00 |  | -0.04 | -0.01 | -0.04 | -0.44 | 0.17 | -1.00 | NC |

^1^ Selection steps are the same as defined in Table S9, where ‘0’ is the original random population without selection. * and ** indicate the significance levels of *p* = 0.05 and 0.01 based on chi-square tests. ‘NC’ indicate the situation where LD’ statistics could not be computed because one or both loci in the analysis reach the fixation.
