# Supplementary material for: Dissecting Genetic Networks Underlying Complex Phenotypes: The Theoretical Framework
Source: PLoS One. 2011 Jan 20;6(1):e14541. doi: 10.1371/journal.pone.0014541 (PMC3024316; doi:10.1371/journal.pone.0014541)
Supplement: Table S11 — Genetic parameters of 16 QTLs in seven groups (QG) affecting plant heights identified in the IR64/Azucena DH population evaluated in 1994 wet season at IRRI [36]. (0.07 MB DOC) [file pone.0014541.s011.doc]

**Table S11.** Genetic parameters of 16 QTLs in 7 groups (*QG*) affecting plant heights identified in the IR64/Azucena DH population evaluated in 1994 wet season at IRRI [36]

| QTLs | QG | Marker interval | Effects (cm) | F | P |
| --- | --- | --- | --- | --- | --- |
| *SD1* | *QG_1_* | RZ730 - RG810 | 12.2 | 395.7 | 2.1x10^-40^ |
| *QPh3b* | *QG_1-1_* | RZ394 - RZ284 | -4.4 | 48.8 | 1.5x10^-10^ |
| *QPh4a* | *QG_1-2_* | RG908 - RG190 | -3.8 | 37.3 | 1.2x10^-8^ |
| *SD1* vs *QPh3b* | *QG_1-1_* | RZ730 vs RZ394 | -5.2 | 62.9 | 1.1x10^-12^ |
| *SD1* vs *QPh4a* | *QG_1-2_* | RZ801 vs RG190 | -3.1 | 24.2 | 2.7x10^-6^ |
| *SD1* vs *QPh2a* | *QG_1-4_* | RZ730 vs RG654 | -3.1 | 23.5 | 3.7x10^-6^ |
| *SD1* vs *QPh7b* | *QG_1-5_* | RZ801 vs RG511 | -4.6 | 47.6 | 2.4x10^-10^ |
| *SD1* vs *QPh12* | *QG_1-6_* | RG810 vs RG457 | -3.1 | 24.0 | 3.0x10^-6^ |
| *QPh8a* | *QG_1-3_* | TGMS1.2 - AG8 | -5.0 | 48.9 | 1.5x10^-10^ |
| *SD1* vs *QPh8a* | *QG_1-3_* | RZ730 vs TGMS1.2 | -4.3 | 36.0 | 2.1x10^-8^ |
| *SD1* vs *QPh9b* | *QG_1-3_* | RZ801 vs RZ422 | 4.8 | 59.6 | 3.3x10^-12^ |
| *Qph8a* vs *QPh9b* | *QG_1-3_* | TGMS1.2 vs RZ422 | -5.3 | 54.6 | 1.9x10^-11^ |
| *QPh3a* | *QG_2_* | RG348 – RZ329 | 4.3 | 15.7 | 3.1x10^-4^ |
| *QPh3c* | *QG_3_* | CDO87 - RG418a | 5.3 | 25.1 | 1.2x10^-5^ |
| *QPh3c vs QPh7a* | *QG_3_* | RG418a vs RG773 | -6.0 | 33.1 | 1.2x10^-6^ |
| *QPh4c* | *QG_4_* | RZ590 – RG143 | 5.1 | 21.5 | 3.9x10^-5^ |
| *QPh9a* | *QG_5_* | C711 – CDO509 | 4.4 | 14.2 | 5.4x10^-4^ |
| *QPh1 vs QPh5a* | *QG_6_* | RG381 – RZ649 | 6.1 | 41.4 | 1.3x10^-7^ |
| *QPh5b vs QPh11* | *QG_7_* | RG13 – RZ536 | 6.7 | 107.5 | 1.6x10^-18^ |

^1^ All QTL main and epistatic effects and associated statistics were obtained using ANOVA. ^2^ All QTL effects of loci in the *QG_2_*-*QG_6_* were estimated by controlling the *SD1* effect in the genetic background of *sd1*. The effect of typical complementary epistasis *QG_7_* was estimated from their theoretical genetic expectation based on Table S8.
