# Supplementary material for: Dissecting Genetic Networks Underlying Complex Phenotypes: The Theoretical Framework
Source: PLoS One. 2011 Jan 20;6(1):e14541. doi: 10.1371/journal.pone.0014541 (PMC3024316; doi:10.1371/journal.pone.0014541)
Supplement: Table S12 — Inferred effects on plant height (cm) of the SD1 mediated downstream pathways (QG1-3, QPh8a and QPh9b) based on the theoretical expectations and observed plant heights (in cm) of the tri-locus genotypes at the corresponding QTLs. (0.06 MB DOC) [file pone.0014541.s012.doc]

**Table S12.** Inferred effects on plant height (cm) of the *SD1* mediated downstream pathway (*QG_1-3_*, *QPh8a* and *QPh9b*) based on the theoretical expectations and observed plant heights (in cm) of the tri-locus genotypes at the corresponding QTLs (Table S6)

|  |  | Multi-locus genotypes ^1^ | | | | | | | | The inferred pathway effect (cm) ^2^ | |
| --- | --- | --- | --- | --- | --- | --- | --- | --- | --- | --- | --- |
| QTL group |  | AABBCC | AABBcc | AAbbCC | AAbbcc | aaBBCC | aaBBcc | aabbCC | aabbcc |  |  |
| *SD1* vs *QG_1-3_* | N | 21 | 21 | 8 | 4 | 25 | 6 | 12 | 13 |  |  |
|  | Mean | 126.5 | 109.4 | 108.7 | 103.1 | 92.0 | 89.1 | 89.9 | 88.8 | $A_{QG_{1-3}}=19.4$ | |
|  | Expectation | $A_{QG_{1-3}}+C_{QG_{1-3}}+C$ | $C_{QG_{1-3}}+C$ | $C_{QG_{1-3}}+C$ | $C_{QG_{1-3}}+C$ | $C$ | $C$ | $C$ | $C$ |  |  |

^1^ Capital letters AA, BB and CC represent the presumed functional alleles of *SD1*, *QPh8a* and *QPh9b* (Table S11) for increased PH, and the small letters are the non-functional mutant alleles of zero effect.

^2^ The inferred pathway effects were estimated from their theoretical genetic expectations of the multilocus genotypes based on **model (2)**.
