# Supplementary material for: Dissecting Genetic Networks Underlying Complex Phenotypes: The Theoretical Framework
Source: PLoS One. 2011 Jan 20;6(1):e14541. doi: 10.1371/journal.pone.0014541 (PMC3024316; doi:10.1371/journal.pone.0014541)
Supplement: Table S13 — Inferred effects of the SD1 mediated QTL groups (QG1 expect for QG1-3), QG3, QG6 and QG7 on plant height based on the theoretical expectations and observed plant heights of the multilocus genotypes at the corresponding loci. (0.15 MB DOC) [file pone.0014541.s013.doc]

**Table S13.** Inferred effects of *SD1* mediated QTL groups (*QG_1_* expect for *QG_1-3_*), *QG_3_* , *QG_6_* and *QG_7_* on plant height based on the theoretical expectations and observed plant heights (in cm) of the multilocus genotypes at the corresponding loci (Table S6)

| QTL |  | Digenic genotypes at the interacting loci ^1^ | | | | Inferred pathway effect (cm) ^2^ | | |
| --- | --- | --- | --- | --- | --- | --- | --- | --- |
| group |  | AABB | AAbb | aaBB | aabb |
|  | N | 36 | 25 | 24 | 26 |  | |  |
| *QG_1-1_* | Mean | 118.7 | 105.2 | 93.1 | 85.9 | $A_{QG_{\text{1-1}}}=13.5$ | | |
|  | Expectation | $A_{QG_{1-1}}+C_{QG_{1-1}}+C$ | $C_{QG_{1-1}}+C$ | $C$ | $C$ |
|  | N | 26 | 35 | 39 | 22 |  | |  |
| *QG_1-2_* | Mean | 121.0 | 108.9 | 90.4 | 90.1 | $A_{QG_{\text{1-2}}}=12.1$ | | |
|  | Expectation | $A_{QG_{1-2}}+C_{QG_{1-2}}+C$ | $C_{QG_{1-2}}+C$ | $C$ | $C$ |
|  | N | 21 | 42 | 28 | 27 |  | |  |
| *QG_1-4_* | Mean | 122.9 | 109.2 | 90.9 | 89.7 | $A_{QG_{\text{1-4}}}=13.7$ | | |
|  | Expectation | $A_{QG_{1-5}}+C_{QG_{1-5}}+C$ | $C_{QG_{1-5}}+C$ | $C$ | $C$ |
|  | N | 43 | 17 | 20 | 39 |  | |  |
| *QG_1-5_* | Mean | 118.7 | 104.5 | 93.3 | 88.9 | $A_{QG_{\text{1-5}}}=14.1$ | | |
|  | Expectation | $A_{QG_{1-6}}+C_{QG_{1-6}}+C$ | $C_{QG_{1-6}}+C$ | $C$ | $C$ |
|  | N | 32 | 27 | 26 | 32 |  | |  |
| *QG_1-6_* | Mean | 118.7 | 109.2 | 92.2 | 89.4 | $A_{QG_{\text{1-6}}}=9.5$ | | |
|  | Expectation | $A_{QG_{1-7}}+C_{QG_{1-7}}+C$ | $C_{QG_{1-7}}+C$ | $C$ | $C$ |
|  | N | 17 | 6 | 18 | 11 |  | |  |
| *QG_3_* | Mean | 79.4 | 99.7 | 96.2 | 92.7 | $A_{QG_{3}}=-16.8$ | | |
|  | Expectation | $A_{QG_{3}}+C_{3}$ | $C_{3}$ | $C_{3}$ | $C_{3}$ |
| *QG_6_* | N | 7 | 16 | 15 | 22 |  |  | |
|  | Mean | 107.3 | 85.2 | 87.9 | 90.4 | $A_{QG_{6}}=19.5$ | | |
|  | Expectation | $A_{QG_{6}}+C_{6}$ | $C_{6}$ | $C_{6}$ | $C_{6}$ |
| *QG_7_* | N | 35 | 21 | 41 | 22 |  |  | |
|  | Mean | 109.8 | 95.7 | 96.3 | 109.0 | $A_{QG_{7}}=13.4$ | | |
|  | Expectation | $A_{QG_{7}}+C_{7}$ | $C_{7}$ | $C_{7}$ | $A_{QG_{7}}+C_{7}$ |

^1^ Capital letters represent the presumed functional alleles of the QTLs (Supplementary Table S 2) for either increased or decreased trait values, and the small letters are the non-functional mutant alleles inferred based on the theoretical expectations of the digenic genotypes of **model (2)**. The Effect of *QG_3_ and QG_6_* was estimated by controlling the *SD1* effect in the genetic background of *sd1*.

^2^ The inferred pathway effects were estimated from their theoretical genetic expectations of the multilocus genotypes based on **model (2)**. The effect of typical complementary epistasis *QG_7_* was estimated from their theoretical genetic expectation based on S8.
