# Supplementary material for: Dissecting Genetic Networks Underlying Complex Phenotypes: The Theoretical Framework
Source: PLoS One. 2011 Jan 20;6(1):e14541. doi: 10.1371/journal.pone.0014541 (PMC3024316; doi:10.1371/journal.pone.0014541)
Supplement: Table S14 — Identification of 19 functional genetic units (FGUs) affecting submergence tolerance (ST) by χ 2 tests (single loci) and multi-locus probability tests in 71 ST introgression lines selected from 1900 BC3F2 plants derived from the cross between NPT (recurrent parent) and Khazar (donor). (0.08 MB DOC) [file pone.0014541.s014.doc]

**Table S14.** Identification of 19 functional genetic units (FGUs) affecting submergence tolerance (ST) by *X^2^* tests (single loci) and multi-locus probability tests in 71 ST introgression lines selected from 1900 BC_3_F_2_ plants derived from the cross between NPT (the recurrent parent) and Khazar (donor)

| FGU ^1^ | Bin ^2^ | Marker | B | H | Gene action | Freq. | *X^2^_2_* | P |
| --- | --- | --- | --- | --- | --- | --- | --- | --- |
| *AG_1_* | 5.6 | RM87 | 54 | 0 | 1 | 0.761 | 1247.7 | 1.2x10^-271^ |
| *AG_1_* | 6.2 | RM314 | 56 | 0 | 1 | 0.789 | 1345.9 | 5.5x10^-293^ |
| *AG_1_* | 9.3 | RM321 | 53 | 0 | 1 | 0.757 | 1218.7 | 2.3x10^-265^ |
| *AG_2_* | 2.6 | RM475 | 8 | 0 | 1 | 0.113 |  | 4.5x10^-365^ |
| *AG_2_* | 5.1 | RM122 | 8 | 3 | 1 | 0.134 |  | 4.5x10^-365^ |
| *AG_2_* | 5.3 | RM437 | 9 | 3 | 1 | 0.148 |  | 4.5x10^-365^ |
| *AG_3_* | 7.1 | RM481 | 3 | 13 | 4 | 0.134 | 25.1 | 3.5x10^-6^ |
| *AG_3_* | 11.6 | RM206 | 14 | 0 | 1 | 0.215 | 75.6 | 3.8x10^-17^ |
| *AG_4_* | 4.2 | RM261 | 9 | 2 | 1 | 0.141 | 23.1 | 9.6x10^-6^ |
| *AG_4_* | 10.2 | RM216 | 7 | 0 | 1 | 0.099 | 14.7 | 6.4x10^-4^ |
| *AG_5_* | 2.5 | RM300 | 12 | 0 | 1 | 0.169 | 48.0 | 3.8x10^-11^ |
| *AG_5_* | 2.11 | RM166 | 10 | 2 | 1 | 0.155 | 30.0 | 3.1x10^-7^ |
| *AG_5_* | 7.4 | RM336 | 13 | 0 | 1 | 0.183 | 57.5 | 3.3x10^-13^ |
|  | 11.1 | RM286 | 32 | 0 | 1 | 0.457 | 420.9 | 4.0x10^-92^ |
|  | 5.4 | RM169 | 27 | 4 | 1 | 0.408 | 303.4 | 1.3x10^-66^ |
|  | 2.7 | RM263 | 19 | 0 | 1 | 0.268 | 133.7 | 9.3x10^-30^ |
|  | 5.5 | RM161 | 4 | 10 | 4 | 0.132 | 13.9 | 9.6x10^-4^ |
|  | 7.6 | RM248 | 8 | 0 | 1 | 0.113 | 19.5 | 5.8x10^-5^ |
|  | 11.5 | RM21 | 16 | 0 | 1 | 0.232 | 94.6 | 2.9x10^-21^ |
|  | 4.6 | RM303 | 12 | 20 | 4 | 0.314 | 162.0 | 6.6x10^-36^ |
|  | 12.6 | RM17 | 25 | 3 | 1 | 0.373 | 252.2 | 1.7x10^-55^ |
|  | 8.3 | RM544 | 15 | 3 | 1 | 0.232 | 56.7 | 4.8x10^-13^ |
|  | 8.6 | RM502 | 14 | 0 | 1 | 0.200 | 69.0 | 1.0x10^-15^ |
|  | 12.5 | RM235 | 8 | 1 | 1 | 0.120 | 18.1 | 1.2x10^-4^ |
|  | 8.3 | RM25 | 14 | 0 | 1 | 0.197 | 67.8 | 1.9x10^-15^ |
|  | 10.6 | RM228 | 35 | 0 | 1 | 0.500 | 509.3 | 2.6x10^-111^ |
|  | 11.4 | RM287 | 23 | 0 | 1 | 0.324 | 203.2 | 7.5x10^-45^ |

^1^ *AG* = a perfect association group, defined as a group of unlinked but highly associated loci, which was detected by the multi-locus probability tests.

^2^ Bins are inferred from the SSR markers in the reference linkage map of the IR64/Azucena DH population in the GRAMENE database (http//:www.gramene.org).
