# Supplementary figures and images for: Dissecting Genetic Networks Underlying Complex Phenotypes: The Theoretical Framework
Source: PLoS One. 2011 Jan 20;6(1):e14541. doi: 10.1371/journal.pone.0014541 (PMC3024316; doi:10.1371/journal.pone.0014541)

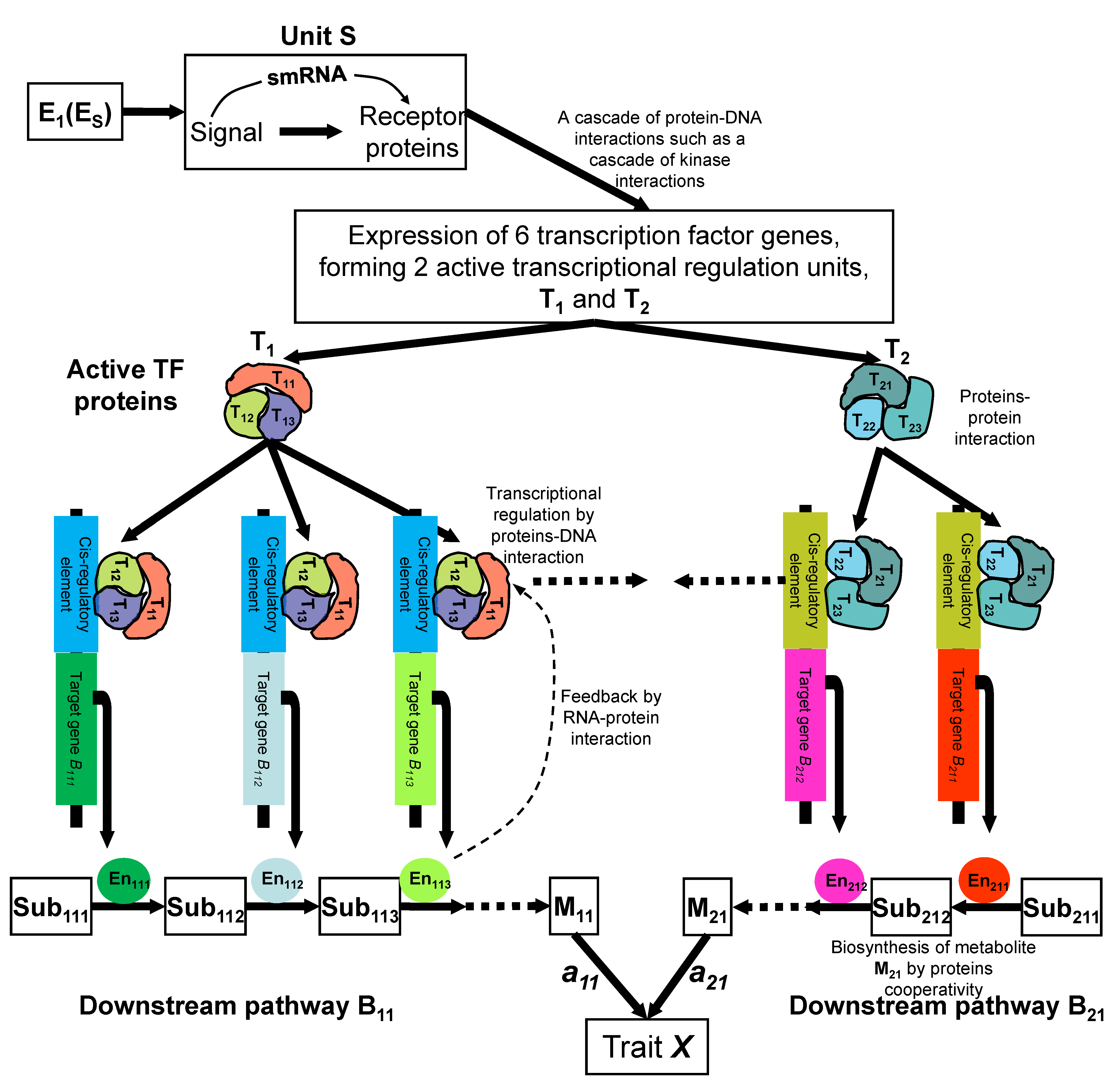

Supplement: Figure S1 — Hypothetical molecular mechanisms involved in a positively regulated signaling pathway affecting trait X, in which a signal from a specific environmental factor, ES, is perceived by one or more receptor proteins either directly or through an smRNA, each encoded by a single gene, forming a single signal transduction (S) unit. The transduction unit then induces the expression of six transcriptional factor genes forming two separate protein complexes, T1 and T2, units. T1 and T2 then each regulate a set of downstream genes B111, B112, B113, etc.; encoding enzymes En111, En112, En113, etc. or B211, B212, etc. encoding En211, En212, etc. that function in downstream pathway B11 or B21, resulting in metabolites M11 or M21, which has phenotypic effect a11 or a21 on trait X. Sub111, Sub112, and Sub113 are biochemical substrates of enzymes En111, En112, En113 encoded by genes B111, B112, B113, respectively. (1.49 MB TIF) [file pone.0014541.s015.tif]

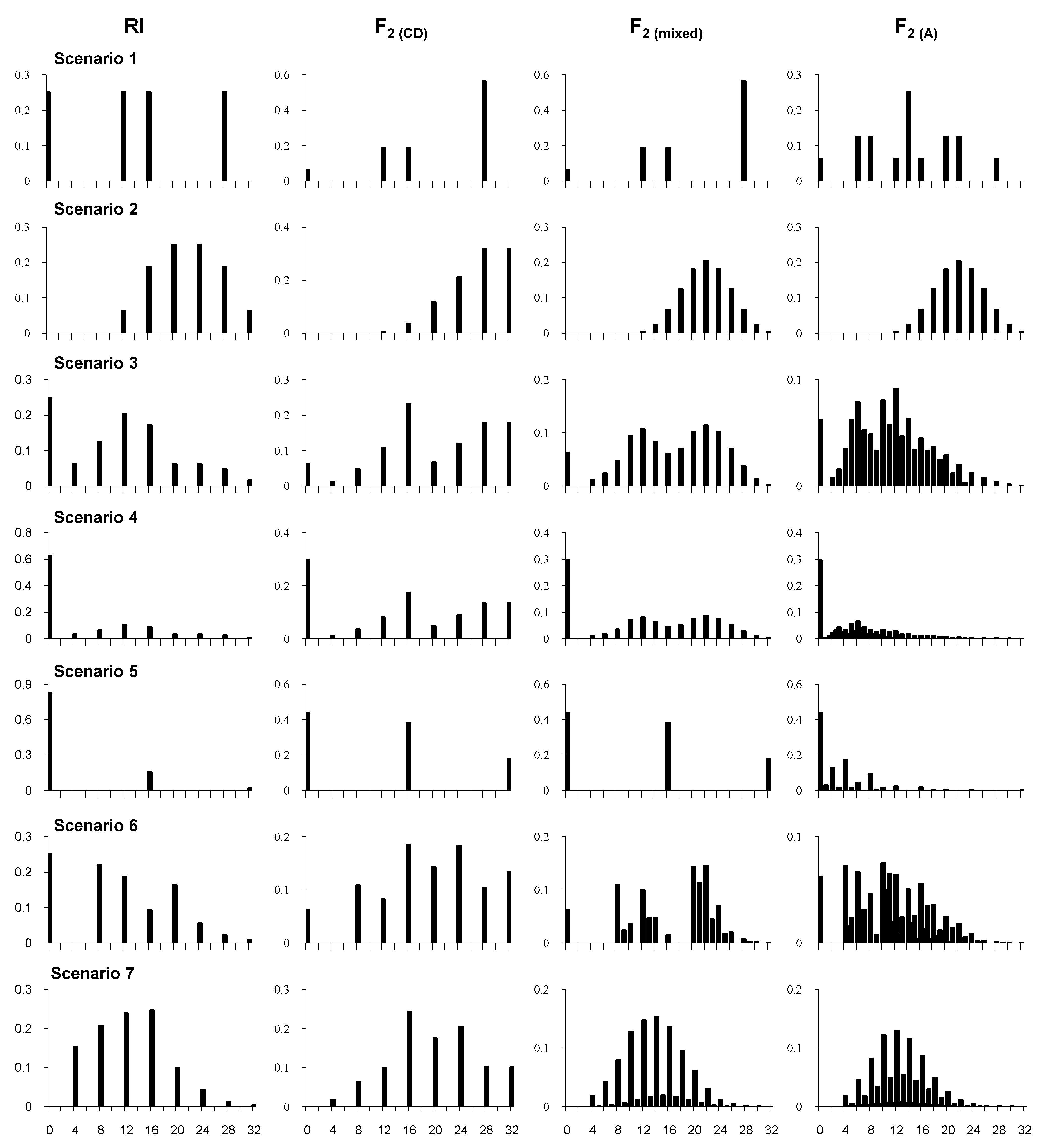

Supplement: Figure S2 — Expected frequency distributions of the phenotypic values of trait X in an F2 and a recombinant inbred line population segregating at different numbers of loci in a single signaling pathway of model (2) under the seven scenarios defined and Table 1 and Figure 1B. (1.13 MB TIF) [file pone.0014541.s016.tif]

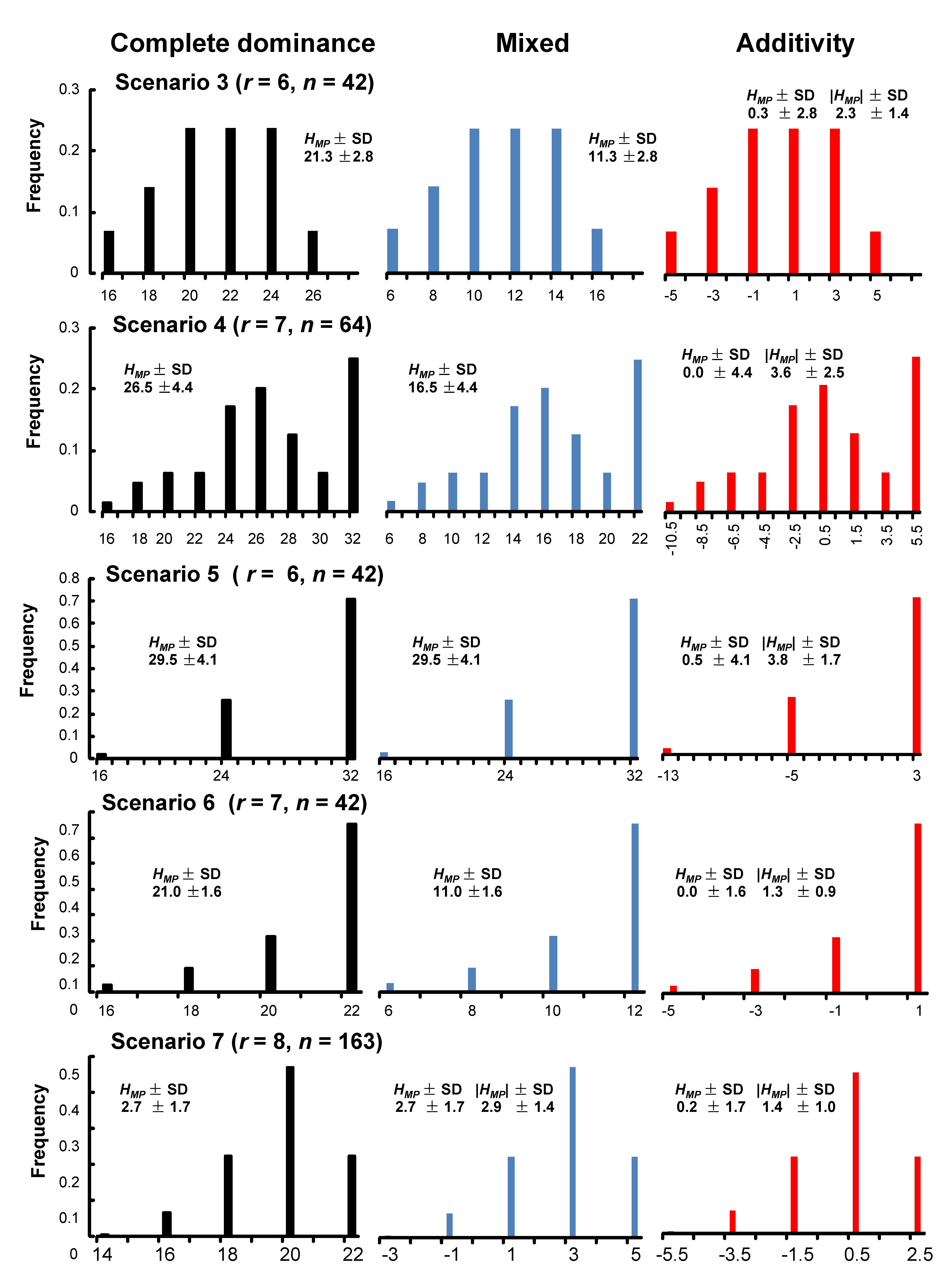

Supplement: Figure S3 — The expected mid-parental trait heterosis (H MP) under three types of gene actions under scenarios 3–7 of Table 1 regarding the type and number of segregating loci in a signaling pathway defined in Figure 1B. In the mixed gene action, all segregating loci at regulatory (S and T) levels are completely dominant, and all loci at the downstream level B act additively. r and n are the numbers of segregating loci and possible distributions of the segregating loci in the parents. (1.64 MB TIF) [file pone.0014541.s017.tif]

**A**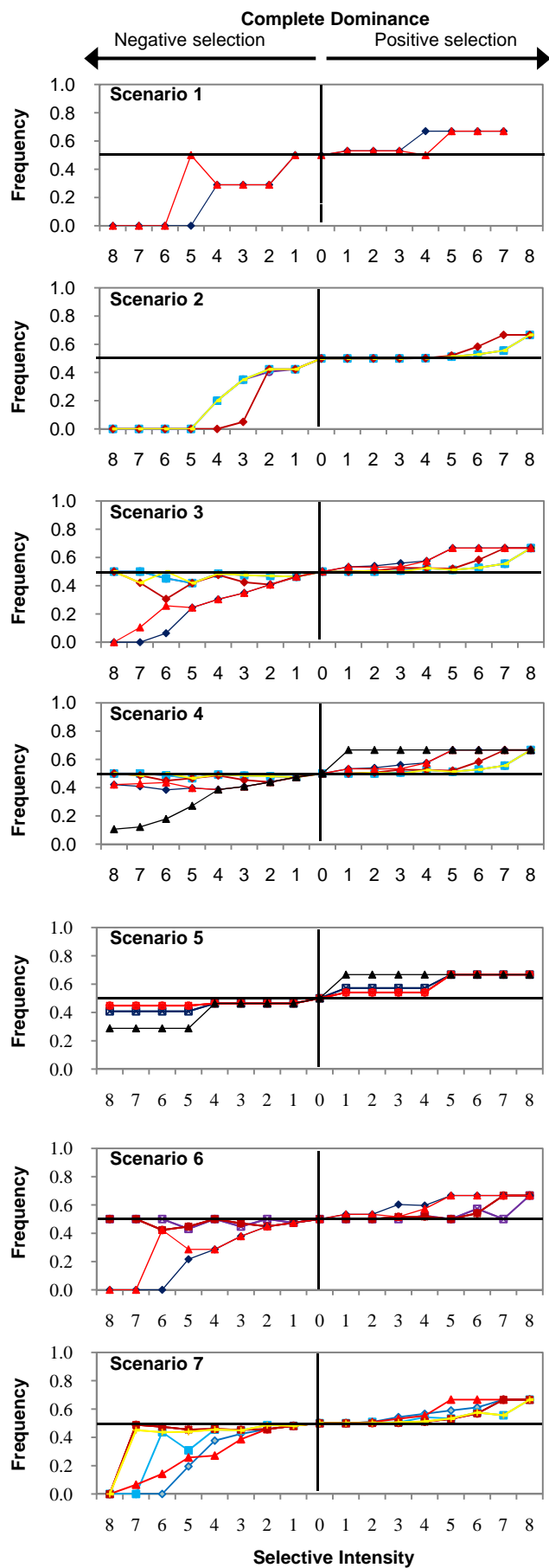**B**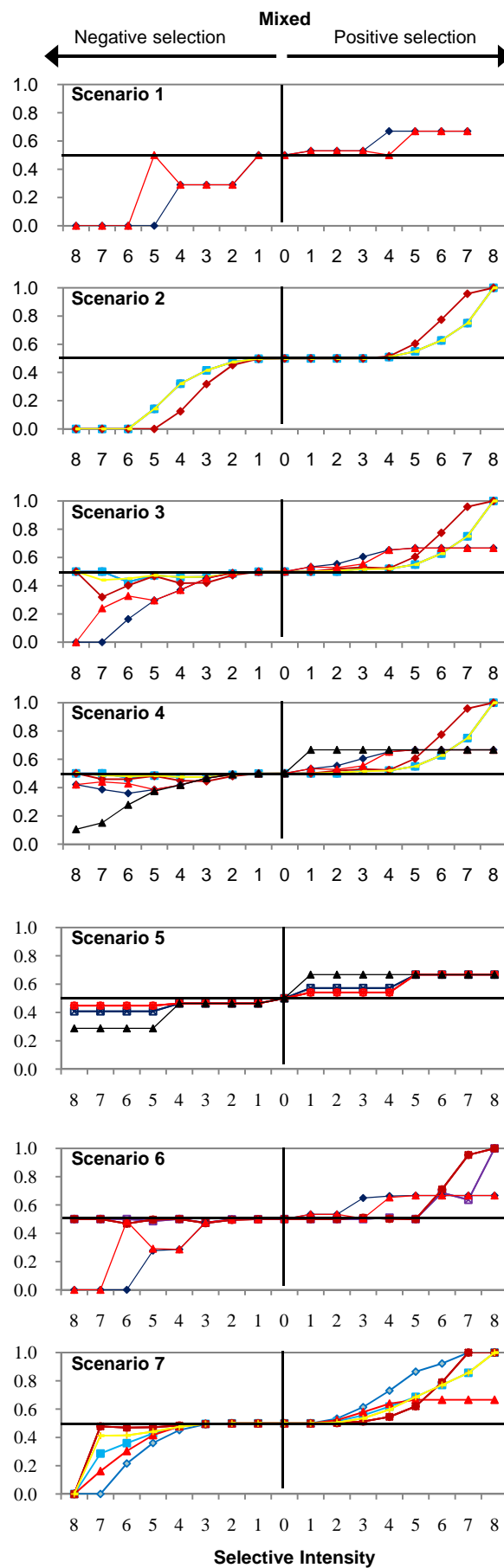

**C**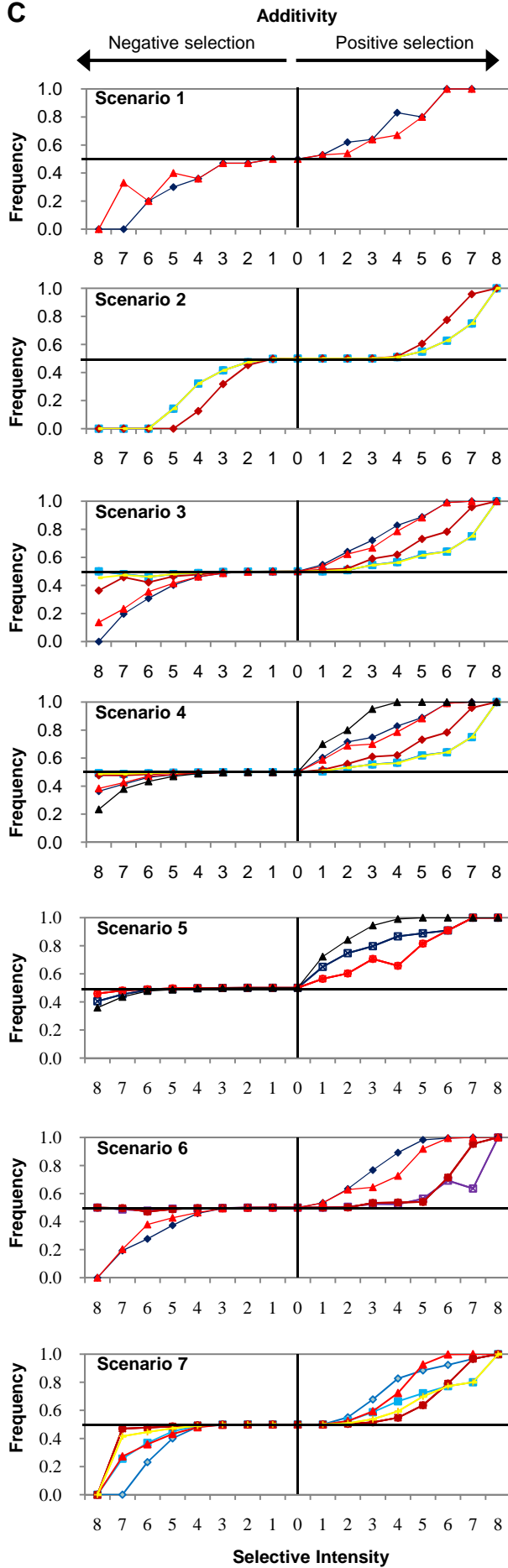**D**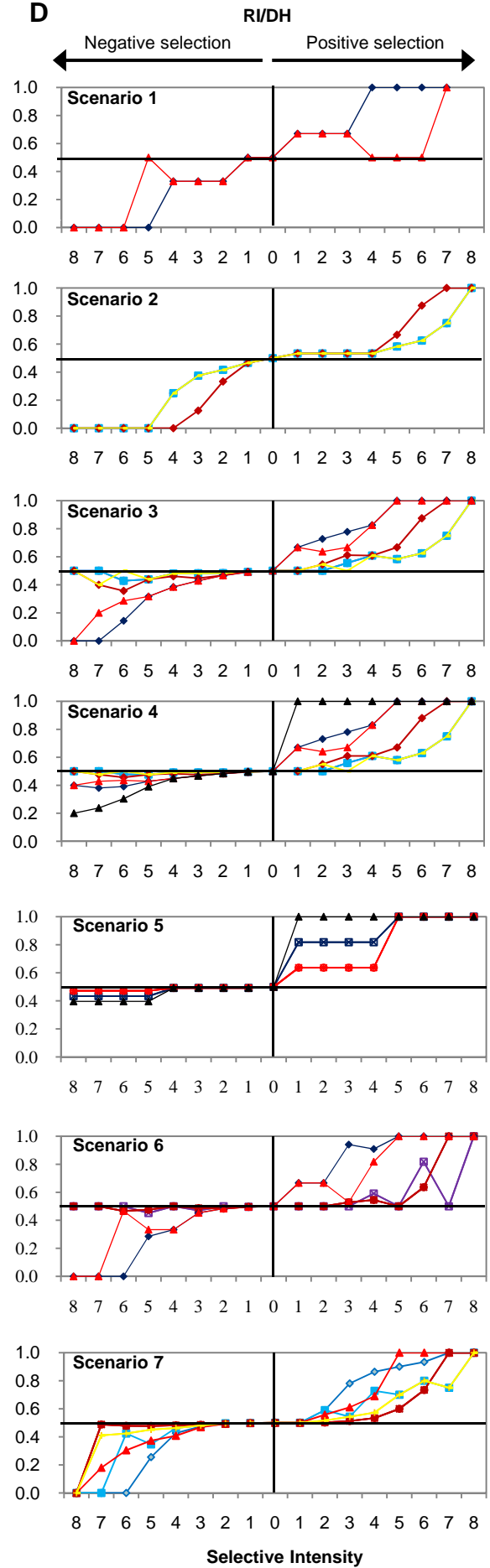

|                                                                                      |                                                                                       |                                                                                       |                                                                                       |                                                                                       |                                                                                         |                                                                                          |
|--------------------------------------------------------------------------------------|---------------------------------------------------------------------------------------|---------------------------------------------------------------------------------------|---------------------------------------------------------------------------------------|---------------------------------------------------------------------------------------|-----------------------------------------------------------------------------------------|------------------------------------------------------------------------------------------|
| 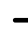 S  | 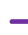 B11 | 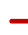 B21 | 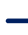 T11 | 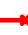 T21 | 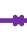 B111 | 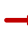 B211 |
| 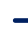 T1 | 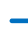 B12 | 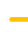 B22 | 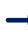 T12 | 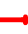 T22 | 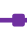 B112 | 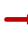 B212 |
| 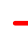 T2 | 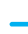 B13 | 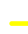 B23 |                                                                                       | 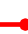 T23 |                                                                                         | 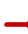 B213 |

Supplement: Figure S4 — The expected cumulated frequency shifts of functional alleles in response to positive and negative selection under the seven scenarios defined in Table 1 (A) under complete dominance at all segregating loci in an F2 population, (B) under mixed gene action (complete dominance for the regulatory S and T loci and additivity for the downstream B loci) in an F2 population, (C) complete additivity in an F2 population, and (D) RI or DH population. In the steps of selection, 1, 2, …, 8 represent the selection trait thresholds of ≥4.0 or ≤4.0, …, ≥32.0 or ≤32.0 for positive or negative selection defined in Table S9. (0.51 MB PDF) [file pone.0014541.s018.pdf]
